# Wedding Amidst War? Armed Conflict and Female Teen Marriage in Azerbaijan

**DOI:** 10.1007/s10680-022-09645-0

**Published:** 2022-10-31

**Authors:** Orsola Torrisi

**Affiliations:** Department of Social Policy, The London School of Economics, Houghton Street, London, WC2A 2AE UK

**Keywords:** Armed conflict, Early marriage, Family formation, Nagorno-Karabakh

## Abstract

Does armed conflict influence female teen marriage? Despite increasing attention to early marriage, its drivers and consequences, quantitative research on whether teen unions are affected by situations of armed violence is minimal. This paper addresses this gap by examining the relationship between exposure to the conflict in Nagorno-Karabakh over 1992–1996 and teen marriage outcomes in Azerbaijan. Using data from the 2006 Demographic and Health Survey and the Uppsala Conflict Data Program, I compare cohorts at risk of teen union before and during the conflict climax years with a modelling strategy that exploits information on forced displacement and spatial variation in conflict violence. Results show that experiencing war violence in adolescent ages, its intensity and frequency are associated with a lower risk of teen marriage. Reductions are largest for the cohorts who spent most of their adolescent ages under conflict and who were displaced as a result. For never-migrant conflict-affected girls, declines extend to the youngest cohorts. The combination of age at conflict occurrence and the experience of disruptive events like forced migration matters for teen marriage outcomes.

**Supplementary Information:**

The online version contains supplementary material available at 10.1007/s10680-022-09645-0.

## Introduction

There are at least three reasons why demographers and policymakers should be concerned with whether armed violence affects early union formation. The first relates to the scale of the issue: globally, over 650 million women alive today—or 1 in 5—are estimated to have married in adolescence, and the highest rates of teen unions, i.e. marriages involving girls aged 12–19, are in countries with great levels of political violence (UNICEF, 2020; 2013). With a growing number of people and children living in conflict-torn contexts, the issue evidently has the potential to impact the lives of increasingly many girls and families worldwide (Østby et al., 2020; UNOCHA, 2019). Second, early marriage is a violation of the Universal Declaration of Human Rights bearing profound and lasting consequences on individuals, e.g. educational and socio-economic disadvantage (Dahl, 2010; Lyngstad, 2006), poor pregnancy outcomes and higher maternal mortality (Ganchimeg et al., 2014; Nove et al., 2014), domestic abuse and union dissolution (Kiplesund & Morton, 2014; Teachman, 2002) and implications for future generations and other aspects of social life, including gender equality and public health (UNICEF, 2005; Nour et al., 2006). Situations of armed violence exacerbate these human and social costs (Mazurana et al., 2019). Third, in many low- and middle-income countries, shifts in union formation are strongly tied to changes in the timing of childbearing, future fertility patterns, and long-term population dynamics. If women marry sooner, *ceteris paribus*, reasonably, their lifetime fertility will raise and contribute to population growth (Onagoruwa & Wodon, 2018). Anticipating similar scenarios is key for post-conflict reconstruction strategies, development, and resource allocation (Duflo, 2005; Thiede et al., 2020).

However, demographic research on the relationship between armed violence and teen marriage is remarkably scarce. This study addresses this lacuna. Specifically, it examines whether women in Azerbaijan who were affected by the Nagorno-Karabakh conflict with Armenia and reached their teens in its climax years (1992–1996) had different early marriage trajectories compared to their non-affected peers and to women who were “at risk” of teen union in the pre-conflict Soviet era.

In theory, the relationship could go either way. War may promote early unions through mechanisms that include the search for economic and/or physical security for girls and their families, nationalist pro-natalist policies, and reinforced gender roles (Neal et al., 2016). Alternatively, armed conflict could induce families to postpone the marriages of their young daughters because of financial hardship, forced migration, and disrupted social networks, among others (Shemyakina, 2013; Staveteig, 2011). The extent of these competing scenarios further depends on pre-existing trends in marital timing and women’s ages at conflict occurrence (Neal et al., 2016).

Net of a recent mixed-methods study on early marriage practices among Syrian refugees in Jordan (Sieverding et al., 2020), quantitative research so far considered overall marriage patterns only and yielded inconclusive answers with regard to the sign, and even to the actual presence of a relationship (e.g. De Walque, 2006; Jayaraman et al., 2009; Khawaja & Randall, 2006; Shemyakina, 2013; Valente, 2011). Inasmuch as this literature provides valuable contributions, the focus on general marriage outcomes overlooks the particular vulnerabilities of young population segments in conflict. Further, most of this evidence relies on time-trend comparisons and rarely studies deal with conflict-related migration.

To tackle these issues, I use data from the 2006 Azerbaijan Demographic and Health Survey and conflict information from the Uppsala Conflict Data Program. I estimate survival models specified with a difference-in-difference logic that exploits data on forced displacement, spatial variation in conflict violence and a cohort specification that accounts for the risk of marrying in teen ages before and during the war. The results provide evidence of a significant and robust negative relationship between conflict, its intensity and frequency, and teen union formation. The largest reductions characterise the cohorts who spent most of their teens under active conflict conditions. Further, findings on response heterogeneity by conflict-related migration suggest displacement as a plausible driver of the lower early marriage levels of these cohorts.

This paper makes a unique contribution to the literature on households’ demographic responses to war and socio-economic turmoil as the first to provide empirical evidence directly on teen marriage. Moreover, unlike other accounts of the demographic consequences of armed violence, the available data and peculiar characteristics of the Nagorno-Karabakh conflict allow to explicitly handle and examine forced migration. Albeit findings cannot be interpreted strictly as causal, the use of a design strategy seeking to isolate as much as possible the impact of conflict represents another improvement to the relatively narrow methodological approaches used until now. The study context is also highly pertinent to the research purpose. Since independence and the onset of the dispute with Armenia, Azerbaijan has reported an increasingly high share of marriages involving teenagers (State Statistical Committee of Azerbaijan (SSC), 2011), and today it has one of the greatest rates of adolescent union in Eurasia (UNFPA, 2012, 2014). Differently from the other handful settings studied previously (e.g. Rwanda or Tajikistan), where conflicts reached a peaceful settlement, Azerbaijan’s case also allows investigating the issue in relation to a conflict that was officially “frozen” until 2020 (Cornell, 2017), when violence re-escalated. The findings are thus of tangible interest for policy in Azerbaijan, and in other turbulent settings where unsettled conflicts have begun to evolve into similar simmering dynamics.

## Armed Conflict and Teen Unions: Background, Theory, and Pathways

Despite growing political and programmatic attention to early unions and women’s vulnerabilities in conflict, knowledge about the influence of armed violence on female adolescent marriage is largely limited to qualitative studies, which tend to suggest conflict-related increases (Kohno et al., 2020; Mourtada et al., 2017; Schlecht et al., 2013). Quantitative research assessing the magnitude and drivers—or even just confirming the existence and direction of the relationship at the population-level—is scarce (Neal et al., 2016). To date, only Sieverding et al. (2020)’s mixed-methods study on Syrian refugees in Jordan examined changes in early marriage associated with armed conflict with solid statistical analyses, finding no evidence of increases.

A handful more studies have at least focused on population-level changes in general marriage patterns associated with conflict, offering mixed results. Some of these analyses document declines in union formation during wartime. For example, Khawaja and Randall (2006) and Saxena et al. (2004) found decreasing marriage rates during the second Palestinian Intifada and the Lebanese civil war, respectively. In both cases, the declines occurred for most women, including girls aged 15–19. Union postponement was also observed during the Bosnian war (Staveteig, 2011), the Rwandan genocide (Jayaraman et al., 2009; Verpoorten & Schindler, 2012) and, at least temporarily, in Cambodia under the Khmer Rouge regime (De Walque, 2006). In the one study on a former Soviet context most similar to Azerbaijan, Shemyakina (2013) showed that women in conflict-stricken areas who attained marriage age during or just after Tajikistan’s civil war were less likely to marry than their non-affected counterparts.

However, other analyses report conflict-related marriage increases, even in the same contexts of some of the studies introduced above. For instance, both Staveteig (2011) and Clifford et al. (2010) noted a faster entry into marriage, especially for young women, during the Rwandan and Tajik conflicts. The discordance in findings is due to different methodologies: while Staveteig (2011) and Clifford et al. (2010) analysed only temporal changes in trends within the whole population, Jayaraman et al. (2009), Verpoorten and Schindler (2012) and Shemyakina (2013) relied on more advanced statistical techniques and, importantly, sought to accurately identify war-affected women. Marriage increases were though also documented in research using finer measures of conflict exposure in Nepal (Valente, 2011; Williams et al., 2012), and in a study on displaced Malian Tuareg (Randall, 2005).

Results from these studies are evidently not univocal, presumably because of different methodologies and limited attention to age-groups, sub-populations (e.g. displaced), and to the impact of secular shifts or other simultaneous factors. Yet, by connecting their findings with existing conceptual frameworks on marital timing, including cost–benefit models (Becker, 1981), economic resource theory (Corno et al., 2020) and marital search models (Oppenheimer, 1988), sociological and life-course perspectives, it may be possible to guide expectations specific to teen unions, and advance suggestions on explanatory pathways.

First, classic cost–benefit marriage models may explain conflict-related increases in adolescent unions (Randall, 2005; Valente, 2011). In times of crisis, marriage may be perceived as a “consumption-smoothing” tool generating economies of scales and thus useful to pool scarce resources and share risk (Fussel and Palloni, 2004; Rosenzweig & Stark, 1989), especially where bride-price is customary (Corno et al., 2020). Since also a substantial number of studies in economics and psychology suggest greater risk-aversion in war-affected individuals (Bellucci et al., 2020; Callen et al., 2014), we may expect conflict-stricken households to opt for early “transactional” marriages to secure financial support for their young daughters, offload family economic responsibilities and extend networks (UNICEF, 2013; Hoogeveen et al., 2011). If conflict hits schooling infrastructures hard, resulting in the permanent drop-out of young cohorts, this alternative may be especially true for the youngest and lowest-educated girls (Cetorelli, 2014).

The costs associated with marriage may though elicit the opposite response: as economic conditions deteriorate, employment and housing options become scant or inadequate (Saxena et al., 2004), conflict-affected families may divert spending from the payment of ceremonies to more immediate needs, e.g. health and relocation expenses, at least in the short-term, or may be unable to afford good-sized dowries (Khawaja & Randall, 2006), leading to expect the postponement of unions that would otherwise occur in early ages (Corno et al., 2020).

In addition to economic uncertainty, war brings about physical risks to which girls are particularly vulnerable and that could increase their marriage risk. Households may expedite marriages to protect girls and their honour from forms of physical harm like rape or abductions (Randall, 2005; Sieverding et al., 2020). At the same time, the hunt for physical safety often entails forced migration. Displacement can split existing couples, delay already organised marriages or disrupt social networks functional to finding partners (Crawford et al., 2015; Hutchinson et al., 2016). From a cohort perspective, the disruption of social networks seems particularly relevant for girls in their early teens when displaced, given that early unions are often facilitated by parental social connections in the local community (Schaffnit et al., 2019) and/or rely on consanguineous (kin) relationships (Sieverding et al., 2020). Reduced chances to marry may also be hypothesised for girls whose physical security is violated during conflicts. For instance, those maimed, injured or raped may be perceived as “less desirable” by potential grooms or may themselves be reluctant to search for partners following conflict trauma (Staveteig, 2011).

Marital search models may further suggest war-induced changes in girls’ marital trajectories resulting from variations in sex-ratio and shortages of men (Warner et al., 2011). If individuals look for partners in specific areas, and the likelihood of union formation is highest when there is plenty of potential mates, mass mobilisation and excess mortality among young men may reduce the availability of suitable partners, leading to marriage declines (De Walque, 2006). Alternatively, sex-ratio imbalances could increase the prevalence of informal or polygamous unions as young unmarried women look for sources of support (Staveteig, 2011). Though, in the study setting most akin to Azerbaijan, Shemyakina (2013) finds no relationship between variation in local sex-ratios during Tajikistan’s war and female age at marriage.

It is also plausible to expect war-induced broad structural changes, including shifting gender dynamics, rising nationalism and the break-down of social cohesion, to alter girls’ marital timing (Neal et al., 2016). Sociological research, for example, suggests that young women’s increased participation to non-traditional roles, e.g. in the workforce or the battlefield, may result in empowerment gains and greater control over life choices, including the deliberate decision to delay marriages (McKay and Mazurana, 2004; Etchart and Baksh-Soodeen, 2005). Alternatively, conflict may reinforce stereotypical gender attitudes and elevate the expectation of female domesticity. Together with pro-natalist narratives encouraging the “need” to maintain a demographic balance with the enemy and “compensate for” conflict losses, these expectations may expose girls to higher social pressure to marry (Chi et al., 2015; Staveteig, 2011). Moreover, war impinges on social embeddedness, i.e. the breadth, depth and extent of social cohesion within a community (Takács, 2005). Resultant reduced social trust can complicate the search for partners, notably in intrastate conflicts (Cassar et al., 2013) and in traditional societies where kin and intra-community are usually harnessed to arrange weddings (Jayaraman et al., 2009).

Finally, the consequences of macro-level events, including war, are known to vary depending on one’s birth cohort (e.g. O’Brein, 2020; London & Wilmoth, 2016). Hence, a nontrivial, yet so far neglected aspect relates to the life-stages, and thus ages when girls experience conflict (Neal et al., 2016). From this cohort or life-course perspective, it seems reasonable to expect conflict-related marriage changes especially for girls in their early teens at conflict onset. Compared to younger children (who may turn teen as war continues), these girls seemingly would have already reached menarche and would be considered “marriageable” (Ibitoye et al., 2017). Moreover, the fact that this group would spend most, if not all its time “at risk” of adolescent marriage under conflict conditions (while older teens would face a shorter “eligibility” window) would make experiencing war at this life stage particularly impactful.

Evidently, the impact of conflict on union dynamics is more complex than it may appear at first: not only there is theoretical and empirical ambiguity on the drivers; even the sign of the relationship is unclear (Neal et al., 2016). For teen unions specifically, although it is plausible to expect armed conflict to alter risk, current knowledge does not allow generating clear a priori hypotheses about which direction this shift may take as the relationship can go either way. It is also unknown whether war at specific early life stages and the type of conflict experience trigger different responses. The overarching aim here is therefore to determine as neatly as possible *whether* conflict, in its spectrum of manifestations, is actually associated with teen union. Then only, to examine specificities, including conflict intensity and frequency, differences by ages during conflict and by experiences of displacement, which may be informative of explanatory processes.

## The Study Context

### Post-Soviet Azerbaijan: Socio-Economic Changes and the Conflict with Armenia

Significant financial deterioration and instability characterised Azerbaijan’s post-Soviet path to regime change (Singh & Laurila, 2011; World Bank, 2005). The transition period was further complicated by the outbreak of conflict violence with Armenia over Nagorno-Karabakh, a mountainous region officially recognised as part of Azerbaijan (UN Security Council, 1993a, 1993b, 1993c, 1993d; UN General Assembly, 2008), but which Armenia regards as an Armenian historical area of residence (HRW, 1994; Cornell, 2001, 2017).^1^

The conflict traces its roots to the last years of the USSR and its structural arrangements. During the Soviet era, the region was granted an autonomous status—the Nagorno-Karabakh Autonomous Oblast (NKAO)—within the then Azerbaijan Soviet Socialist Republic, but its borders contained a sizable Armenian population (USSR Population Statistical Collect, 1988; de Waal, 2004). When the Soviet centre-dominated control system crumbled, tensions mounted in NKAO and demonstrations reclaiming Nagorno-Karabakh’s membership to Armenia extended from Stepanakert/Khankendi (the capital of NKAO) to Yerevan (de Waal, 2004). Violent rallies causing casualties took place also around Baku.

Confrontational politics turned into outright conflict in December 1991 when, with Armenian support, NKAO proclaimed independence from Azerbaijan (HRW, 1992, 1994). Although disagreement between sources exists on the exact start and end dates of the hostilities, most analysts and official sources indicate early 1992 as the beginning of the full-blown war, 1992–1994 as its most violent period (Fig. A1, Appendix A), and the post-1994 armistice years (1995–1996) as a “cooling-off” phase still characterised by instability, attacks on civilians and conflict-related population movements (HRW, 1992, 1994; CSCE, 2012; ICG, 2005; Cornell, 2015; Huseynov, 2010; Krüger, 2010).

Since then, the conflict has been described as “frozen” (Bebler, 2015; Cornell, 2017).^2^ The resultant de facto Republic of Nagorno-Karabakh (also known as Artsakh), the Western parts of three other officially Azerbaijani districts (Agdam, Fizuli and Terter) and the region of Kelbajar-Lachin became entirely populated and controlled by ethnic Armenians. Altogether these territories comprise approximately 20% of Azerbaijan’s internationally recognised territory (Racz, 2016). Only the Eastern segments of Agdam, Fizuli and Terter remained under Azerbaijan’s jurisdiction as parts of what, in Azerbaijani language, is known as the Upper-Karabakh (Yuxarı-Qarabağ) region (UN Security Council, 1993a, 1993b, 1993c, 1993d).^3^

**Fig. 1:**
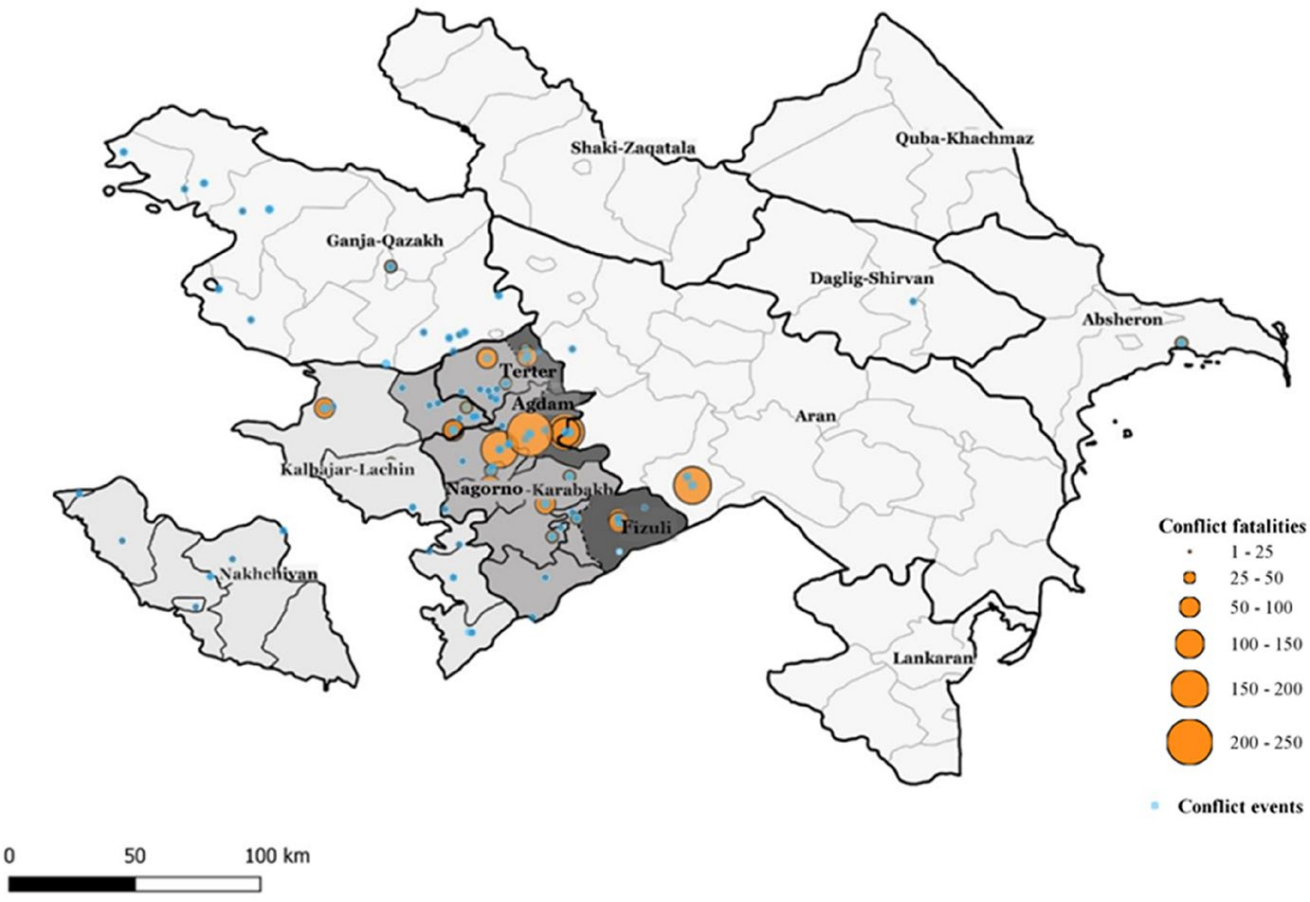
Map of conflict events and fatalities in Azerbaijan 1992–1996, *Source* UCDP-GED (2020). Notes: The map shows the 9 economic regions of mainland Azerbaijan (66 districts) and the exclave of Nakhchivan (7 districts). The non-sampled Nakhchivan and Kelbajar-Lachin economic regions are highlighted in light grey; the de facto Republic of Nagorno-Karabakh (also known as Republic of Artsakh, in 2006 under full-Armenian control and populated only by ethnic Armenians) and the sampled parts of the contested districts of Agdam, Terter and Fizuli (Upper-Karabakh) are, respectively, in progressively darker grey. Blue dots indicate conflict events. Larger orange dots denote increasingly high number of conflict fatalities as measured by UCDP-GED best estimate. Multiple conflict events occurred in the same location, so blue dots sometimes overlap

An estimated 17,000–25,000 Azerbaijani died in the conflict (HRW, 1994; de Waal, 2004; Yunusov, 2002). No official or consolidated gender/age-disaggregated estimate is available either for civilian or for military deaths.^4^ However, some evidence suggests that the killing of civilians and other atrocities, like rape and torture, occurred indiscriminately on both sides, and that Azerbaijani military losses were predominantly males (Amnesty International, 1993; HRW, 1994; UNDP, 2007).^5^ The conflict further imbued an already patriarchal society with a nationalist rhetoric celebrating male fighters as heroic “martyrs” and valuing women for their roles of wives and mothers of future defenders (Twum et al., 2019).

Antagonistic nationalism was fuelled by the plight of displacement. The conflict caused the mass expulsion of *all* ethnic Azerbaijani from Armenia and Nagorno-Karabakh. Although exact numbers are contested, over 750,000 Azerbaijani—seemingly equally divided by gender (UNHCR, 2009)—had to relocate to safer areas within Azerbaijan and were granted *prima facie* internally displaced person (IDP)/refugee status (CSCE, 2017; HRW, 1994; IDMC, 2007; IOM, 1997; UNHCR, 2003, 2009, 2015). Because of this heavy inflow, about 10–15% of the country's then total population of 8 million, for years Azerbaijan was the country with the largest per capita number of IDPs in its national population (Greenway, 2009; UNHCR, 2009). As of 2016, one in 15 Azerbaijani was still displaced and none lived in Nagorno-Karabakh (UNHCR, 2017).

### Marriage Traditions and Early Unions in Azerbaijan

Marriages are central to Azerbaijani culture and have important socio-economic functions (Tohidi, 1999). The formalisation of unions involves large spending for celebrations and expensive financial transactions, including the dowry paid by the bride's family (*cəhiz*), the bride payment made by the groom (*başlığ*) and other inter-families material exchanges. This borrowing and lending of currency and assets then serve to enact social status and expand networks (Yalçın-Heckmann, 2001).

For these reasons and the social stigmatisation of late marriages or singlehood, early marriages were common in pre-Soviet Azerbaijan (UN Azerbaijan, 2015; Havilov, 1991). In the Soviet period, however, rates declined sharply thanks to several measures targeting Islamic and customary marriage practices, including bans on child marriage, polygamy, arbitrary divorce, and to mandatory schooling for women (Edgar, 2006; Heyat, 2014; Lapidus, 1978).

Since independence in 1991, and even after the 1995 legal prohibition to contract marriage before 18, official figures have reported an increasing share of marriages involving adolescents (SSC, 2011). These numbers are likely an undercount since official statistics only include marriages registered at State agencies, whereas unions involving adolescents tend to be first celebrated with unofficial religious ceremonies (*kəbin*) and formally registered once the youngest spouse (typically the bride) reaches the legal marriageable age (UNFPA, 2014).

## Data and measures

### Data

The primary data source for analysis is the 2006 Azerbaijan Demographic and Health Survey (AZ-DHS), which collected various demographic, family and conflict-related information from a nationally representative sample of a total of 8,444 Azerbaijani women aged 15–49 years.^6^ The sample was generated in two stages: clusters were first selected in Baku and Azerbaijan’s other administrative units using the 1999 Population Census as sampling frame. Households were then listed in each cluster and systematically selected, with an overall response rate of 98%. For security reasons, the sample excluded the Nakhchivan exclave and, due to their contested status, the Kelbajar-Lachin economic region and the Western parts of Agdam, Fizuli and Terter (Fig. 1). In 2006, these latter were de facto controlled and only populated by ethnic Armenians (Nagorno-Karabakh National Statistical Service, 2006), not the focus of this paper*.*

**Table 1:** Cumulative probabilities of teen marriage by birth cohort (1957–1984)

3-year birth cohort	Mean marriage age	Median marriage age	Age first married (%)						*N* (weighted)
			15 and below	16 and below	17 and below	18 and below	19 and below	Not married by 20	
1957–1959	21.78	21	1.29%	3.71%	10.42%	21.89%	31.14%	68.86%	525
1960–1962	22.51	22	0.56%	1.81%	6.58%	16.53%	24.40%	75.60%	767
1963–1965	22.04	22	1.40%	1.73%	7.51%	16.77%	27.55%	72.45%	779
1966–1968	21.43	21	0.98%	2.88%	9.35%	20.46%	31.17%	68.83%	702
1969–1971	21.91	21	0.39%	1.11%	6.41%	14.71%	29.82%	70.18%	696
1972–1973	21.22	20	0.67%	1.87%	9.82%	22.41%	39.56%	60.44%	403
**1974–1977**	**20.79**	**20**	**3.18%**	**8.91%**	**18.36%**	**28.50%**	**38.90%**	**61.10%**	**804**
**1978–1980**	**20.16**	**20**	**3.33%**	**10.18%**	**16.99%**	**25.62%**	**35.33%**	**64.67%**	**665**
**1981–1984**	**19.68**	**20**	**2.14%**	**5.82%**	**12.84%**	**19.80%**	**27.67%**	**72.33%**	**1,073**
*Total obs*									6,414

*Source* 2006 AZ-DHS. *Notes* *N* indicates the total number of women in the sample weighted using provided sample weights. Cohorts of women who reached teen ages during the conflict years are highlighted in bold

*The second data source I employ is the* Uppsala Conflict Data Program Georeferenced Event Dataset (UCDP-GED), which openly provides worldwide spatial and chronological coordinates on conflict episodes and casualties (Croicu & Sundberg, 2016). Its data are widely used in research and judged to be of the highest quality available for this study’s aims (Eck, 2012).

### Variables

Information on the timing of marriage come from the AZ-DHS women’s questionnaire, which asks respondents: *“In what month and year did you start living with your (first) husband/partner as if married?”*. Since weddings involving adolescents can go unregistered until spouses grow older, the AZ-DHS employs this wording to effectively capture women’s date of marriage rather than its registration. I use this question to construct my dependent variable and analyse “survival” time to teen marriage. Drawing from international definitions and past empirical studies (WHO, 2015; UNICEF, 2020; Dahl, 2010), teen marriage is defined as unions involving girls aged 12–19. Other individual-level socio-demographic information, including year of birth, residence location/type, also come from the AZ-DHS.

I construct several variables to identify women affected by the war and thus to determine changes in teen union associated with conflict. The first is a cohort-level measure, coded as one for the cohorts turning 12–19 during the conflict because these women were “at risk” of teen union in wartime. The second measures *overall* conflict-affectedness, combining information on self-reported displacement status and residence in conflict-stricken districts in Upper-Karabakh. This indicator is intended to capture women’s conflict experiences in their spectrum of manifestations. Finally, using UCDP-GED data, I construct two supplementary continuous variables that help delving into the relationship with war *intensity* and *frequency*. Table A1 (Appendix A) summarises these variables and their mutual relationships. Each is described in detail below.

#### Cohort-Level Exposure to Conflict

The first conflict measure is based on women’s ages in wartimes and thus on birth cohort. Since the conflict could have only influenced the teen marriage decisions of those aged 19 and below at its onset, I generate a cohort variable based on women’s entry into/exit from the pool of marriageable adolescents and their ages between 1992 and 1996 (Shemyakina, 2013; O’Brein, 2020). Table A2 (Appendix A) identifies relevant cohorts by showing women’s age at conflict onset (1992), after it peaked and ended (1996), the year in which they “started” (turned 12) and “ceased” (turned 19) to be eligible for teen union, and their age at survey time. Figure A2 (Appendix A) shows akin information in a corresponding a Lexis graph format.

Women aged 21 + at conflict onset (born 1957–1971) were teenagers *before* the war and the USSR dissolution; hence, they were too old to have their teen marriage outcomes influenced by the conflict. I call this group the “*Soviet cohort”*. Conversely, women who turned 12–19 between 1992 and 1996 (born 1974–1984) were “at risk” of teen marriage during the peak conflict years. I define this group as the “*War-cohort*”.

Later, I further disaggregate this latter group into women who spent their *late* (born 1974–1977), *almost entire* (1978–1980) or *early* (1981–1984) teens under conflict to examine differences across early life stages.

#### Overall Conflict-Affectedness Indicator

The second measure—the *overall conflict-affectedness* indicator—is constructed by combining three groups of conflict-affected women into a single binary variable, exploiting their location when the conflict began, which the AZ-DHS allows to retrace despite lacking full migration histories.

First, unlike most household surveys, the AZ-DHS asks all respondents aged 16 + two separate questions about IDP/refugee status.^7^ If an interviewee identifies as refugee or IDP from Nagorno-Karabakh, s/he is then asked about the country or district s/he moved from as a result. These questions permit the identification of a first conflict-affected group: Azerbaijani women who lived in Armenia or Nagorno-Karabakh when the conflict erupted, *and* who also experienced resultant forced displacement.

Second, the questionnaire asks about years lived in the current place of residence. I use this information to identify a second group of conflict-affected respondents: women who always resided (or migrated pre-conflict, i.e. before 1992) in the Upper-Karabakh region, namely in Azerbaijan-controlled and sampled areas of Agdam, Fizuli and Terter (Torrisi, 2020). These women were not forced out of their territories and, perhaps, their specific villages did not suffer from major disruptions. However, these were still affected by conflict events (Fig. 1). Importantly, due to residential proximity to the core conflict zones and the contested status of their districts, these women likely faced recurring indirect exposure and subtle conflict-related insecurities (e.g. fear of coercive acts, land expropriation), with potential consequences for family-related decision-making. This group also includes few non-IDP/refugee women (*n* = 54) who migrated to these districts during conflict years.

Lastly, I identify a third group: non-refugee/IDP women with at least one male member of their natal household (e.g. father, brother) or the mother, if she was the household head, who declared being displaced by the war. In the initial phase of the exodus, indirect registration costs (e.g. travel to registration points) were presumably high, while food allowances were granted to families, provided that their head was a registered IDP/refugee (UNHCR, 2009; ICG, 2012; Kalin et al., 2010). Although the survey was implemented sufficiently after to make-up for any initial under-registration and Azerbaijan’s government granted practically universal protection to citizens fleeing the conflict, the above reasons still do not exclude that some conflict-affected women went unregistered (and hence unreported). This coding procedure tackles this potential source of underreporting.^8^

By combining women affected by conflict both directly (experienced violence and displacement) and indirectly (through physical proximity, or having conflict-affected family members), the *overall conflict-affectedness* indicator serves as a starting point and measures the experience of conflict in its possible manifestations. In subsequent analyses, I separate its specific components to learn about potential heterogeneity, including differences by displacement status.

#### Conflict intensity and frequency indicators

I complement the discrete indicator with two supplementary continuous variables for *frequency* and *intensity* of conflict, using UCDP-GED event and fatality data.

Several studies examining conflict effects on other outcomes exploit the UCDP-GED georeferenced nature and link the dataset directly with geolocated survey clusters (e.g. Østby, 2020). Unfortunately, the AZ-DHS did not gather fine-grained GIS cluster data that would allow similar procedures and only provides numerical information on women’s current district of residence. Therefore, we know their economic region of residence (e.g. Aran, Absheron), but only the numeric code of their specific district in that region.^9^ However, the AZ-DHS allows to trace back IDPs’ origin district before they fled Nagorno-Karabakh. We also know that women in Upper-Karabakh resided in the sampled parts of either Agdam, Fizuli or Terter. I thus creatively exploit UCDP-GED data and link them to the groups used to construct the binary indicator.

I do so in a sequential manner. First, I map the exact location of all conflict events and related fatalities occurred between January 1992 and December 1996 as recorded by the UCPD-GED. Figure 1 shows their spatial distribution. About 81% of events (blue dots) and almost all casualties (orange dots) occurred in Agdam, Fizuli and Terter or areas characterised by complete forced migration (Nagorno-Karabakh and Kelbajar-Lachin), thereby allowing to capture conflict intensity and frequency with a good degree of accuracy. Second, I calculate the district-level number of conflict episodes (*frequency*) and fatalities per 1,000 population as per the 1989 USSR Population Census (*intensity*) between 1992 and 1996. Third, I match the computed values to the groups earlier identified as “conflict-affected”.

Based on their origin district in Nagorno-Karabakh, I assign the specific district-level values of each continuous indicator to IDP women (and to women with an IDP/refugee household member as described above). I assign to refugees from Armenia the average value of conflict events and deaths occurred across all districts in Nagorno-Karabakh because we do not know where these women lived in Armenia and hence the exact extent of violence they experienced there. The values are similar to the number of conflict episodes and fatalities that occurred across conflict-affected districts of Armenia. Finally, permanent residents of Upper-Karabakh districts (Agdam, Fizuli, Terter) are assigned averages of conflict events and fatalities that occurred in these three districts between 1992 and 1996. For the few women who migrated to these districts during the conflict, I calculate the same measures, but starting with the year they arrived rather than 1992. For instance, the mean number of conflict events across the three districts in Upper-Karabakh was 13 between 1993 and 1996 and 9 between 1994 and 1996. If a woman moved to these districts in 1993, she is considered exposed to 13 events; to 9 if she moved in 1994. All other women, including non-IDP/refugees in districts affected by some conflict events in otherwise relatively peaceful regions, e.g. Ganja-Qazakh, are considered as affected by no events/fatalities. I address this potential measurement error in the robustness checks.

## Empirical Strategy

To study the relationship between conflict and teen marriage, I estimate complementary log–log (cloglog) survival models. These are here preferred to standard OLS regressions because of the time-to-event nature of the outcome variable and because they allow accounting for censoring of the observations and exit from the risk-set at different times for each subject. Further, I chose a cloglog link function because the survey records duration data in discrete units, and the probability of the event is small. The cloglog model is also the discrete-time analog of a proportional hazard model and thus coefficients, once exponentiated, can be interpreted as hazard ratios (Allison, 1982). In the models, exposure to the risk of teen marriage starts at age 12 for all women and ends on the date of teen marriage. Women who had not married in their teens are censored just before their 20th birthday.

I adopt a difference-in-difference (DID) *logic* that leverages on variation in conflict-affectedness across cohorts and space (i.e. where respondents lived at the time of the war). In its simplest form, the DID design envisages two populations and two time points. In the first period, both populations are exposed to the same conditions. In the second, a “treatment” unrolls in one population (“treated”), but not in the other (“control/comparison”). Following this standard language, conflict is here to be thought as the “treatment” condition. The design of this paper slightly differs from the traditional DID in its time component: rather than using a pre-/post-“treatment” time-period variable, I rely on cohort variation, i.e. I compare conflict-affected women who turned 12–19 during the conflict years with their not-affected peers and women aged 21 + at conflict start (thus no longer “eligible” for teen marriage). This is because the main goal is to focus on teen ages, and conflict peaked in specific years; once these are fixed, the only variation comes from women’s year of birth. This strategy also allows to fully harness the survey retrospective nature in lack of pre-/post-conflict rounds.

Equation 1 presents the basic statistical framework for empirical analysis:

1 $$\begin{aligned} \log {\text{ }}({-}\log {\text{ }}(1{-}\pi _{{{\text{ikdt}}}} )){\text{ }}\, =\, & \alpha _{t} + {\text{ }}\gamma \;{\text{Conflict}}_{{\text{i}}} + \beta ({\text{Conflict}}_{{\text{i}}} \times {\text{War}} - {\text{Cohort}}_{{{\text{ik}}}} ) \\ ~ & + {\text{ }}\theta _{{\text{k}}} + {\text{ }}\lambda _{{\text{d}}} + ~\upsilon _{i} + \varepsilon _{{{\text{ikd~}}}} \\ \end{aligned}$$

where *π*_ikdt_ is the conditional probability of teen marriage at interval *t* for woman *i* in cohort *k* in district *d* at wartime, provided that she has not already married. *War-Cohort*_ik_ indicates women turning 12–19 during conflict. In the main specification, *Conflict*_*i*_ is the binary *overall conflict-affectedness* indicator. The coefficient *β* of the interaction term identifies the relationship between being affected by conflict and the probability of entering teen marriage in the *War-cohort*. In alternative specifications, I relax the binary indicator into the continuous frequency (events) and intensity (fatalities) conflict indicators.

*α*_t_ is the duration function indicating how risk depends on time (effect of age on the hazard) and is specified by breaking the hazard function into *n* categories (< 5 years, 5–6 years and so on) during which the risk of the outcome is assumed constant for women with the same pattern of covariates. *θ*_k_ and *λ*_d_ are birth-year and district dummies, respectively. These control for the underlying trend in teen unions due to belonging to an older versus a younger cohort and for time-invariant local conditions affecting marriage patterns independent of conflict. Models also adjust for residence type. Given endogeneity (women marrying earlier tend to leave school prematurely), the main models do not control for education. Finally, I add a “frailty” term υ_i_ at the individual level, which allows for unobserved heterogeneity. This term is interpreted as the residual between-women variance due to unmeasured time-invariant attributes that might influence one’s “susceptibility” to marriage, but that cannot be accounted, e.g. women’s parental education/wealth at wartime or union characteristics like arranged/forced marriages (South, 2001; Uecker & Stokes, 2008; Wiik, 2009). This is analogical to individual fixed effects in standard panel data models. Moreover, “frailty” prevents a biased estimation of the coefficients due to the “premature” exit from of subjects whose omitted characteristics make them at “high-risk” of the outcome (Jenkins, 1995). Regressions are estimated using sampling weights, and standard errors are clustered at the primary sampling unit level.

In the main models, I exclude cohorts aged 19–20 in 1992 (born 1973–1972) as their “conflict-affectedness” status is less clear-cut: some of them might have married during the conflict, but they were not affected by violence during most of their adolescence. The USSR breakup possibly “contaminated” their marriage prospects more than the conflict itself. I address this issue in the checks. I also exclude respondents married before age 12 (< 1%) and those aged 19 and below in 2006 because of right censoring on the outcome variable. Following these restrictions, the weighted sample comprises women aged 22–49 in 2006 (*N* = 6,011), i.e. born between 1957 and 1971 and 1974–1984. Table A3 shows sample descriptive statistics.

Any causal interpretation and the accuracy of the estimates rely on the assumptions that trends in teen unions would have been the same across the *War* and *Soviet* cohorts in the absence of conflict and that there were no omitted time-varying effects associated with the conflict indicators. I test the plausibility of these assumptions, including balance of covariates and placebo tests, as much as data allow in Appendix B. Lastly, it is worth re-emphasising that conflict-due migration was largely involuntary and universal (all ethnic Azerbaijani in Nagorno-Karabakh/Amenia were expelled from their home territories), displacement was the main form of internal migration during the years of turmoil (international emigration concerned mainly ethnic Russians and Armenians (Aliyev, 2006; Allahveranov et al., 2012; Rowland, 2004)) and IDP/refugee status was granted *prima facie* by the Azerbaijan government to persons fleeing their homes due to the war. Return was not possible, and expellees were culturally and ethnically akin to non-movers and to residents in non-conflict areas.^10^ Unfortunately, no data source allows examining mortality during the flight and related selection in survival. However, the above features should free the operationalisation of the conflict indicators and the estimates from other serious selectivity issues. “Frailty” terms correct for the selective impact of unobserved factors, and I dedicate special attention to heterogeneity by displacement status.

## Results

### Descriptive Analyses of Entry Into Teen Marriage

Table 1 presents measures of central tendency and the cumulative probability of being married by ages 15 to 19 for women born 1957–1984. This includes women who reached their teens during more stable Soviet years (born 1957–1968), who did so partially during the first years of socio-economic instability (1969–1973) and women attaining adolescent ages almost entirely during the conflict and post-Soviet early transition period (1974–1984).

Some interesting patterns arise: first, the mean and median ages at marriage are higher for the 1957–1971 cohorts reaching teen ages in a more stable macro-economic and social environment. The decrease in measures of central tendency characterising younger cohorts seems attributable to a rising proportion of girls marrying in teen ages. For instance, the share of girls born in 1974–1977 married by age 16 and 17 is, respectively, almost 8% and 12% points higher compared to the 1969–1971 cohorts. The same proportions are about 7% and 9% larger than for women born just before (1972–1973). Very similar increases characterise the 1978–1980 cohort. This latter group shows the highest proportion of married by age 15 (3.33%) and 16 (10.18%). Thus, there appears a pattern of earlier entry into union for the cohorts reaching teen ages in the precarious conflict and independence period.

Second, among these women, the proportion unmarried by age 20 steadily increased. For instance, while 61% of the 1974–1977 cohort were still single at age 20, the share was about 4% and 11% points higher in the 1978–1980 and 1981–1984 cohorts, respectively. Seemingly, the “rush” to marry was more prevalent in older *War-cohorts* and only occurred at the youngest teen ages (15–17) for those born after 1977.

**Fig. 2:**
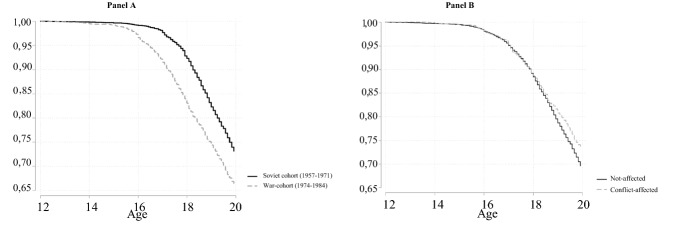
Kaplan–Meier curves for teen marriage by cohorts (Panel A) and conflict-affectedness (Panel B), *Source* 2006 AZ-DHS. Author's own calculation

The Kaplan–Meier curves in Fig. 2 describe these patterns more succinctly and with greater focus on conflict-affectedness. Differences between the *Soviet* and *War-cohorts* are irrelevant until age 15 (Panel A). By age 16, though, the curves start diverging, with a slower entrance at all following ages for the *Soviet cohort*. The largest gap is between ages 17–18 (8 vs. 17%).

The faster entry into marriage of the *War-cohort*, however, is only one part of the story. Not all women born in the 1974–1984 decade were affected by violence in Nagorno-Karabakh. Kaplan–Meier estimates for teen marriage by conflict-affectedness show very little difference between the groups (Fig. 2, Panel B). Only after age 18 the curves marginally separate: conflict-affected women marry slightly later than the non-affected.

**Fig. 3:**
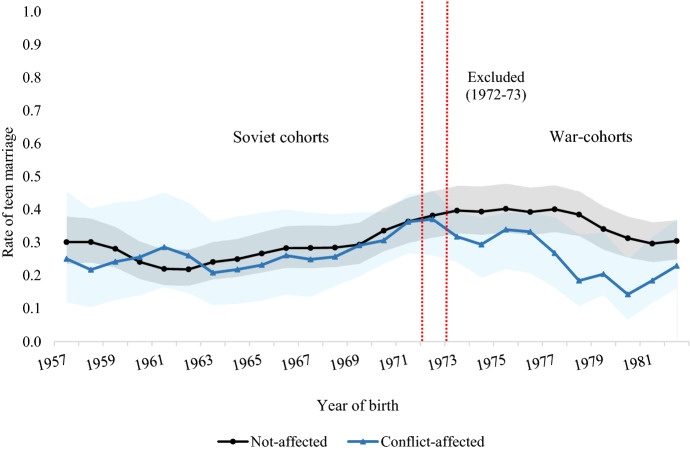
Trends in teen marriage by conflict status and cohorts, Source: 2006 AZ-DHS. Author's own calculation. Shaded areas indicate 95% confidence intervals

As the above descriptions do not supply a univocal picture of the conflict/cohort relationship, I graphically investigate trends in teen marriage rates by birth cohort and overall conflict-affectedness. On the left-hand side of Fig. 3 are rates by conflict-affectedness for the *Soviet cohorts*, namely women who were too old at conflict onset to have their chances of marrying in adolescence affected by violence. On the right, rates for women in the *War-cohorts* who were either affected or not by the conflict. Trends for women in the *Soviet cohorts* with differential exposure to conflict are similar and generally move in parallel. Conversely, there is a wider divergence in the *War-cohorts*: the non-affected have higher and broadly stable teen marriage rates, whereas those of their conflict-affected peers follow a marked, albeit fluctuating, declining pattern. This visual inspection thus suggests a peculiarly different behaviour for women enduring conflict during adolescence compared to both non-affected peers and older women. It also alleviates concerns linked to diverse pre-war marriage trends between groups differently affected by the conflict, thereby reinforcing the logic of the modelling strategy, whose results I discuss next.^11^

### Survival Models

Table 2 shows estimates of survival models specified with a DID *logic* in exponentiated form (hazard ratio, HR). Coefficients greater/lower than one denote a higher/lower risk of teen union compared to the reference category and represent the instantaneous hazard of teen marriage. The hazard is proportionate at any given instant and also cumulative over time. The first two columns report the results of the baseline specification without controls, except duration dependence (Col.1), and adjusted estimates (Col.2) for the main independent variable.

**Table 2:** Results of discrete-time cloglog models of the transition to teen marriage

	HR of teen union			
	(1)	(2)	(3)	(4)
War cohort (1974–1984)* conflict measure	0.659*	0.635*	0.983*	0.942*
	[0.45,0.97]	[0.43,0.94]	[0.97,0.99]	[0.88,0.98]
*Overall conflict-affectedness (ref: Not-affected)*				
Conflict-affected	1.001	1.268		
	[0.72,1.39]	[0.74,2.17]		
Conflict events			1.037*	
			[1.00,1.07]	
Conflict fatalities per 1,000				1.093
				[0.96,1.24]
District dummies	No	Yes	Yes	Yes
Year of birth dummies	No	Yes	Yes	Yes
Controls	No	Yes	Yes	Yes
*σ*_u_^2^	1.559	1.127	1.141	1.121
*N person-years*	44,885	44,885	44,885	44,885

*Source* 2006 AZ-DHS. Notes: Sample consists of women born 1957–1984 (aged 22–49 in 2006), excluding women born 1972–1973. Subjects enter analysis at age 12. Columns represent hazard ratios. 95% confidence intervals are in parentheses. Robust standard errors clustered at the PSU level. The “War-cohort” includes women born 1974–1984. The binary indicator “overall conflict-affectedness” is equal to “1” for IDP/refugee women, non-migrant women residing in Upper-Karabakh and non-displaced women with at least one male member of their family of origin (or mother) who identified as IDP/refugee, and “0” otherwise. All regressions control for duration since start of exposure to the risk of teen marriage (< 5 years, 5–6 years and > 6 years) and rural/urban residence and include a constant not shown. Models are specified with individual-level frailty terms (*σ*_u_^2^) and are weighted using provided sampling weights. *p < 0.05, **p < 0.01, ***p < 0.001

Both models reveal a significantly negative coefficient estimated on the interaction between *War-cohort* and *overall conflict-affectedness*: the risk of teen union is about 34% points lower (Col.1: HR 0.659, 95% C.I. 0.447–0.972) for affected women born in 1974–1984 compared to their non-affected peers and older women (with the same unobserved characteristics). The sign and magnitude are similar when controls are included (Col.2). The minor amount of variance due to unobserved woman-level characteristics suggests that reductions for the conflict-affected do not simply result from selection due to unobserved factors.

**Fig. 4:**
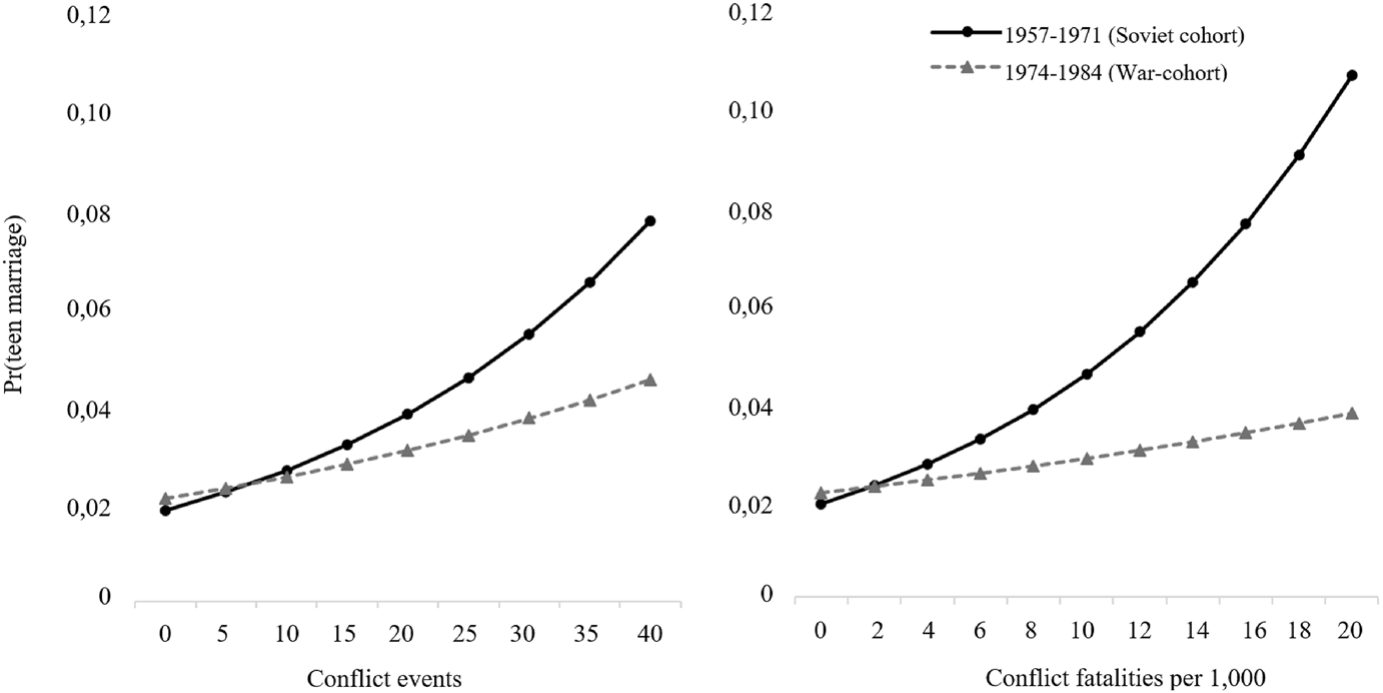
Predicted probabilities of teen marriage by conflict-affectedness frequency and intensity, *Source* As per Table 2, Column (3) and (4)

When I use the continuous conflict frequency and intensity measures, results confirm a significant negative association (Col.3–4). For instance, one standard deviation increase in district fatalities (2.5 casualties) lowers teen marriage risk by about 14% in the *War-cohort*. The continuous measures have similar coefficient sizes and trajectories due to their strong correlation and can be visualised in Fig. 4. While the predicted probability of entering union in teen ages is approximately the same for women in the *War-cohort* and *Soviet cohorts* who did not experience any violence, it increases much less rapidly for the former as the number of conflict events and fatalities increases. The coefficient of *conflict frequency* is positive and significant, denoting that intense violence occurred in areas with higher levels of teen marriage.

### Alternative Measures and Robustness Checks

Results are robust to various checks. First, I restricted the *Soviet cohort* to include only women aged 21–31 in 1992 (born 1961–1971). At the detriment of sample size, this makes this group as close, and therefore, as comparable as possible to the *War-cohort*. Estimates do not change substantively (Table B4). The coefficient size is now larger for all conflict indicators, strengthening the finding of a negative association.

Second, I run Eq. (1) including the 1972–1973 cohorts in the sample. Women born in 1972 were aged 20 at conflict onset, while the 1973 cohort was 19. Initially, I code both as belonging to the *Soviet cohort*. Next, I split them so that the former is assigned to the *Soviet cohort* and the latter to the *War-cohort*. The direction and size of the relationship remain unchanged in both specifications (Tables B5–B6). In the first model, though, the reduction is stronger (*p* < 0.01) for the binary and frequency indicators, suggesting that the largest differences emerged for women aged 18 or below at the start of the full-blown war.

Third, I recoded the continuous conflict measures into three categories for “No events/fatalities”, “Medium” (between one and the 95^th^ percentile, i.e. 24 events and 5.6 fatalities) and “High” (above the 95^th^ percentile). Estimates show that the reduction was essentially driven by medium frequency of exposure and high-intensity violence (Table B7).

Fourth, I estimated models excluding non-displaced women residing in the Ganja-Qazakh region, where a few conflict events also took place. Results are unchanged with respect to the main models, except for the conflict frequency measure (not shown). Here, the relationship is still negative, but is no longer significant (*p* = 0.08). Estimates did not change when I coded as not-affected non-IDP/refugee women with an IDP/refugee member of their origin families, when I excluded them or dropped refugees from Armenia from the sample (not shown).

Models estimated with a logit-link function, alternative specifications of duration dependence (e.g. quadratic, cubic), cut-offs for early unions (e.g. survival time to marriage from 12 to 16/18 to focus on the earliest ages at marriage), shorter conflict time-window (1992–1994/95), including an education dummy (completed mandatory 9-years of schooling) and exposure to media sources did not yield different results.

Due to data availability, the approach taken here is not that of a traditional DID. Hence, performing its entire battery of sensitivity tests was not feasible. However, the robustness of the findings to different specifications, thresholds and definitions, and the checks presented in Tables B1–B3 are reassuring as for the validity of the main results.

### Heterogeneity

#### Does Age at Conflict Matter?

Determined the presence and sign of the relationship, the next relevant question concerns whether all conflict-affected women in the *War-cohort* experienced systematic declines, or if these were limited to specific cohorts and hence ages at conflict. Reasonably, we could expect the strongest relationship for girls who spent most of their time “at risk” of teen union under conflict conditions, i.e. those aged 12–14 at conflict onset (16–18 at denouement). I therefore re-estimated the models using a finer cohort measure which spells out the relationship for women born in 1974–1977 (aged 15 + in 1992), 1978–1980 (14–12) and 1981–1984 (11–8).

**Fig. 5:**
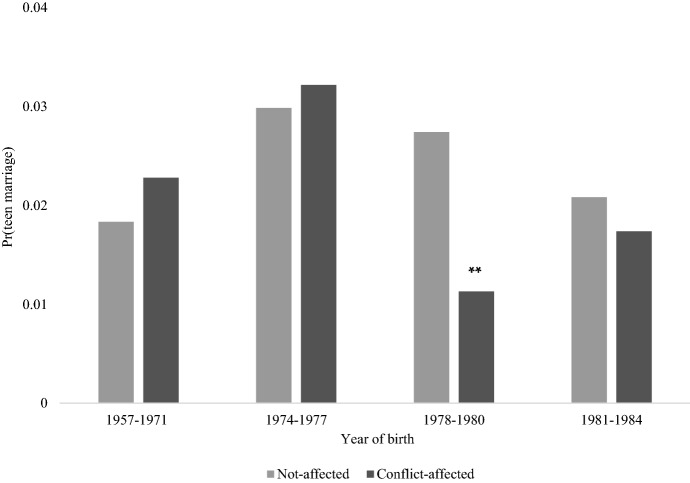
Predicted probabilities of teen marriage by conflict-affectedness and granular cohorts, *Source* As per Table 3, Column (1). *p < 0.05, **p < 0.01, ***p < 0.001

Results in Table 3 show a lower risk of teen union for all *War-cohort* subgroups, though the reduction is significant only for the hypothesised 1978–1980 cohort (HR: 0.327; 95% C.I. 0.156–0.689). No differences in risk characterise women who experienced conflict predominantly in their late teens or childhood.^12^ To aid interpretation, Fig. 5 shows predicted probabilities for each combinations of the interaction term from Col.1.

**Table 3:** Results of discrete-time clog-log models of the transition to teen marriage by granular cohorts

	HR of teen union		
	(1)	(2)	(3)
Conflict measure * Born in			
1974–1977	0.866	0.992	0.969
	[0.50,1.49]	[0.97,1.00]	[0.92,1.02]
1978–1980	0.327**	0.958*	0.846*
	[0.16,0.69]	[0.92,0.97]	[0.74,0.96]
1981–1984	0.668	0.986	0.958
	[0.37,1.20]	[0.96,1.01]	[0.87,1.06]
*Overall conflict-affectedness (ref: Not-affected)*			
Conflict-affected	1.246		
	[0.73,2.12]		
Conflict frequency (events)		1.037*	
		[1.00,1.07]	
Conflict intensity (fatalities per 1,000)		1.107	
			[0.94,1.30]
District dummies	Yes	Yes	Yes
Year of birth dummies	Yes	Yes	Yes
Controls	Yes	Yes	Yes
σ_u_^2^	1.181	1.152	1.134
*N person-years*	44,885	44,885	44,885

*Source* 2006 AZ-DHS. Notes: Sample consists of women born 1957–1984 (aged 22–49 in 2006), excluding women born 1972–1973. Subjects enter analysis at age 12. Columns represent hazard ratios. 95% confidence intervals are in parentheses. Robust standard errors clustered at the PSU level. The “War-cohort” includes women born 1974–1984. The binary indicator “overall conflict-affectedness” is equal to “1” for IDP/refugee women, non-migrant women residing in Upper-Karabakh and non-displaced women with at least one male member of their family of origin (or mother) who identified as IDP/refugee, and “0” otherwise. All regressions control for duration since start of exposure to the risk of teen marriage (< 5 years, 5–6 years and > 6 years) and rural/urban residence and include a constant not shown. Models are specified with individual-level frailty terms (*σ*_u_^2^) and are weighted using provided sampling weights. *p < 0.05, **p < 0.01, ***p < 0.001

The different early marriage behaviour of affected and non-affected girls born 1978–1980 suggests that in Azerbaijan, entry into teen unions was neither immediately manipulated by families as a response to conflict threats, nor the impact extended to cohorts attaining adolescent ages towards the later stage of the war. Rather, the negative association characterised only those in their early teens at conflict onset and thus who were for longest “eligible” for teen marriage under conflict conditions.

#### The Role of Forced Migration

**Fig. 6:**
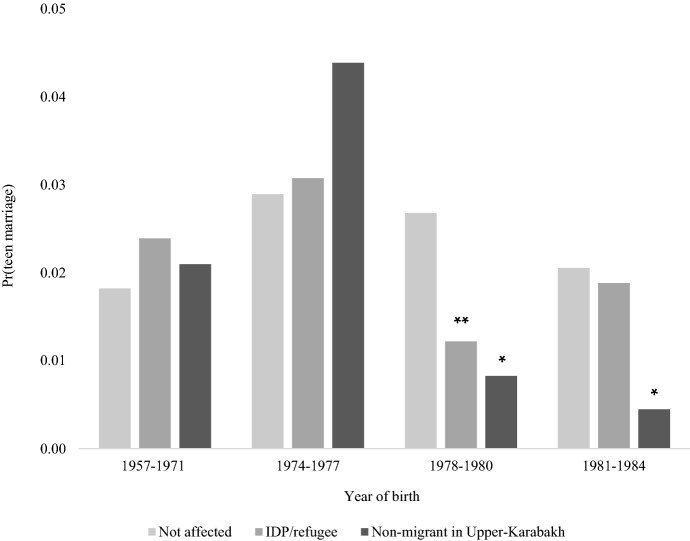
Predicted probabilities of teen marriage by conflict-related migration status and granular cohorts, *Source* As per Table A4 (Appendix A), Column (1). *p < 0.05, **p < 0.01, ***p < 0.001

Several underlying forces may explain the lower levels of early marriage for the 1978–1980 cohorts. One is forced migration. For these girls, displacement occurred precisely in ages when they (and their families) would be more likely to take a decision about teen marriage and search suitable spouses. Conceivably, their displacement and resultant disruption in livelihoods/social networks hindered union formation. In contrast, younger displaced girls had seemingly more time and relatively more stable conditions (e.g. in tent settlements with better access to social and economic assistance) to meet future grooms before actually becoming “at risk” of teen marriage. Slightly older women in the *War-cohort* could have had their marriages already arranged before the conflict and, perhaps, sought to relocate to areas near to or with their prospective husbands. I test these hypotheses by adding an interaction between each 3-year *War-cohort* and women’s conflict-related migration status, spelling out the categories of the *Conflict-affectedness* indicator. Figure 6 presents predicted probabilities of teen marriage from the model (Table A4 for full estimates).

The probability of teen marriage is quite low and similar across groups in the *Soviet cohorts*. Non-significant increases characterise all women aged 18–15 at conflict onset (1974–1977 cohorts), particularly non-migrants of Upper-Karabakh. Teen marriage probability drops sharply for forcibly displaced women born in 1978–1980, whereas no significant changes mark their non-affected counterparts. As hypothesised, the decline in the displaced group is limited to this cohort and does not “spread” onto the following one.^13^

For non-migrants in Upper-Karabakh, there are significant reductions in teen marriage for the 1978–1980 cohorts that further extend to girls who began to be “at risk” of the outcome towards the end of the conflict. Their chances of becoming teen brides are close to zero. Although this finding may be due to small cell numbers, other mechanisms may explain the peculiar behaviour of this group, including a short supply of male partners. Unfortunately, similar sex-ratio factors cannot be tested with available data.^14^^,^ 15

## Limitations

While the finding of a reduction in unions is clear and robust to various measurements and checks, examining teen marriage outcomes disaggregated by conflict subgroups and cohorts exposes the research to the estimation risks inherent to small samples. Moreover, the cross-sectional character of most AZ-DHS variables, and limited access to other data sources, prevented examining many theoretically plausible mechanisms. This is regrettable especially for sex-ratio factors and known determinants of early unions such as parental/household characteristics (Kohno et al., 2020; Pesando & Abufhele, 2019). The selective impact of the latter is accounted for by “frailty” terms included in the models.

Although the study sought to thoroughly exploit the depth and breadth of available data, the lack of GIS cluster information and migration histories for all women may have created measurement errors in the conflict indicators. The use of multiple conflict measures and the fact that intense violence occurred in Nagorno- and Upper-Karabakh should limit this concern. It is also worth noting that I cannot fully exclude social desirability bias and misreporting of displacement status. However, several factors reduce concerns over status over/under-reporting (EGRIS, 2018) including (i) my coding procedure (including as conflict-affected women with a displaced person in her natal household), (ii) the survey aims (not linked in any way to direct refugee/IDP assistance), (iii) the question used to identify forced migrants and (iv) the generally neutral attitudes towards displaced persons found in the Azerbaijani context (UNHCR, 2009). Moreover, I found no evidence of evidence of marriage age heaping and/or age displacement across conflict-related variables that could be of concern (Chantler, 2012). This issue is also attenuated by the survey question used to capture union formation.

Finally, estimates are based on a sample of survivors residing in Azerbaijan in 2006, and there is no direct way to determine whether teen marriages were underestimated because of survival bias.

Overall, results should be interpreted carefully as a first attempt at providing answers to questions on *whether* and *how* violence is associated with early unions. Future research should strive for causal assessment and expand this line of inquiry into the “*whys”*. To confirm causality and investigate specific driving pathways though further efforts in developing new tools or refining existing ones, e.g. oversampling conflict-torn populations and including conflict-sensitive questions in surveys, are inevitably required (Bruck et al., 2016).

## Discussion and Conclusion

*Does exposure to armed conflict influence teen marriage?* Existing knowledge on this paramount question either comes from qualitative research unsuited to evaluate population-level relationships or is extrapolated from quantitative studies focusing on changes in general marriage outcomes, not early unions (Neal et al., 2016). These latter analyses examine a few contexts, with a narrow set of methodological approaches that often hide differences across ages and conflict experiences. The resultant evidence is largely inconclusive and therefore of limited assistance to policy.

This study tackles this knowledge gap and provides a first empirical test of the link between war and early marriage. Findings reveal that in Azerbaijan, experiencing war in adolescence was associated with reductions in teen unions, principally for girls who spent most of their teens under active violence and, among them, forced migrants. Conflict intensity and frequency were also linked to lower marriage risk.

These results echo findings from prior studies that investigated the broader conflict/marriage nexus in settings with similar conflict typology (Khawaja & Randall, 2006) and institutional framework to Azerbaijan (Shemyakina, 2013). As for coefficient size, the magnitude is comparable to changes in marriage law raising the minimum marriage age in the Americas (Bellés-Obrero & Lombardi, 2019; Bharadwaj, 2015), but seemingly larger than weather shocks (Corno et al., 2020). Additionally, results are likely a lower bound of the true effect since the conflict erupted in full in 1992, but tensions emerged in the late 1980s.

A decline in teen marriage for conflict-affected girls is a welcome and, perhaps, unexpected result considering suggestions from qualitative accounts. However, some caution in interpreting and generalising this finding is warranted as the slowdown in teen marriage coincided with an antithetic general increase in the Azerbaijani population compared to the Soviet period. The results, therefore, subsume two kinds of differences: one *between* the Soviet and War-cohorts; the other *within* the War-cohorts. The first likely captures the diverse socio-economic incentives and family regimes the *Soviet* and the *War-cohorts* experienced when teens. The former lived under a system where financial stability, security, family-related services, and regulations were arguably provided by the State; conversely, the *War-cohorts* reached adolescence as such value, economic and legal system collapsed. For the non-affected among them, early marriages reasonably represented a source of stability against these swift socio-economic setbacks, a response observed in other ex-Soviet Central Asian countries (Agadjanian & Makarova, 2003; Clifford et al., 2010; Dommaraju & Agadjanian, 2008). The second difference then captures the extra variation *within* the War-cohorts due to the additional insecurity generated by conflict. Ergo, the final result is to be understood as a combination of experiencing the conflict *as well as* the transition to a new socio-political regime.

These findings provide new evidence on family formation decision-making in times of violence and in relation to different stressors and sources of insecurity. Formally testing explanatory mechanisms was not possible due to data constraints. I nonetheless sought to disentangle associations by cohort and advance some speculations on driving channels. Since reductions occurred essentially in a single conflict-affected cohort group, women’s life-stage at war onset and length of time spent “at risk” of teen marriage under conflict conditions seem to matter more than the experience of violence itself. This result further highlights the importance of applying cohort/life-course lenses when studying the consequences of macro-level events.

Since the delay was particularly pronounced for displaced girls, there is reason to think that forced migration in specific life-stages constituted a pathway for marriage postponement. In the earliest phases of displacement, forced migrants incurred in significant unplanned and emergency expenditures, e.g. relocation travels, that, along with low income-generating opportunities and deteriorated housing conditions, strained their economic welfare (SORGU and World Bank, 1995; IDMC, 2007; Gureyeva-Aliyeva & Huseynov, 2011). As a result, these families perhaps could not afford the expected wealth transfers occasioned by weddings, including finding suitable housing for prospective couples (Saxena et al., 2004), and opted or were forced to divert their limited resources on investments other than marriages that were not required to non-affected households (Sieverding et al., 2020).

Moreover, forced migration from Nagorno-Karabakh and Armenia separated extended households and disrupted community ties (Amnesty International, 2007; UN Commission on Human Rights, 1999). At least in the initial post-displacement years, this sudden social fragmentation and loss of intangible assets perhaps frustrated the search for potential spouses of displaced families and girls then “suitable” for marriages. As conditions stabilised, new networks of support and norms of reciprocity between neighbours who were strangers prior to displacement possibly favoured again partner selection *and the arrangement of weddings. This element could partially explain the lack of impact on IDPs/refugees born after 1980.*

*The sharp declines in unions for the youngest non-migrant cohorts* suggest comparable, but longer disruptive changes on the social fabric due to conflict. A tentative explanation, that cannot be addressed with present data, relates to imbalances in sex ratio. Conceivably, conflict-caused high male mortality and conscription imposed structural changes to the local marriage market of Upper-Karabakh, lowering the amount of available prospective husbands (De Walque, 2006). Although Shemyakina (2013) did not find any relationship between declines in marriage and sex-ratio in Tajikistan, a country that experienced conflict around the same time and with socio-cultural backgrounds comparable to Azerbaijan, similar mechanisms should not be discarded and represent an important avenue for future research.

Delaying marriages from teen to more adult ages, even by a few years, is a desirable outcome for Azerbaijan and girls in violent contexts. This finding though does not exclude adverse marriage outcomes from happening just a bit later than in adolescence. In humanitarian emergencies, young men’s inability to afford bride price, their conscription, and excess mortality could reduce match quality, leading women to marry older or less educated men (Grabska, 2012; Sommers et al., 2011). Wide spousal age and educational difference are known predictors of marital dissolution (Burazeri et al., 2005) or domestic violence (La Mattina, 2017; Mabsout & Van Staveren, 2010). The share of conflict-affected born in 1974–1984 eventually marrying a man aged 10 + years older in the AZ-DHS is more than double than older women (16 vs. 6%). Together with changing marriage timing, conflict possibly constrained women’s choices via a deteriorated pool of potential husbands.

Policy intervention should consider all these aspects. As conflict-induced declines in early unions imply that young women will depend for longer on their families and/or own resources, it is critical to ensure access to learning opportunities that can make girls prospectively less reliant on future partners, or less acquiescent to unwanted marriage arrangements, which may present slightly later in their life course. Widening learning opportunities would have broader positive spill-over and intergenerational effects. Above and beyond conflict, though, we need concerted policy and research efforts to tackle the rooted socio-cultural acceptance of unwanted early marriage and to effectively implement legal frameworks for child and adolescent protection where, as in Azerbaijan, its prevalence is high.

## Supplementary Information

Below is the link to the electronic supplementary material.

Supplementary file1 (DOCX 316 KB)

## Data Availability

Data supporting the findings of the study are available from the corresponding author on request or, alternatively, can be downloaded in raw format from the DHS Program (https://dhsprogram.com/what-we-do/survey/survey-display-279.cfm) and the UCDP websites (https://ucdp.uu.se/country/373).

## Footnotes

1 This section seeks to summarise as neutrally as possible the conflict’s chronological developments using available official sources and documents. In no way I compare human rights violations on either sides or suggest any specific stance on the dispute.

2 The term ‘frozen’ conflict defines a post-conflict situation where active armed fighting has ceased, but contenders have not reached a stable peace agreement. Although in 1994 an armistice halted open combat, it was not accompanied by a peace deal.

3 There is substantial debate over the names given to the region and adjacent districts (Broers, 2019, p. 85) for a comprehensive discussion). From here and throughout, I use the term “Nagorno-Karabakh” to refer to all the territories under the control of Armenian-supported separatists until survey time, i.e., the de facto Republic of Nagorno-Karabakh/Artsakh (which encompasses the Western parts of Agdam, Fizuli and Terter) and the Kelbajar-Lachin economic region. I employ “Upper-Karabakh” to refer to the Eastern parts of Agdam, Fizuli and Terter (Fig. 1 for more).

4 Estimates from Azerbaijan’s government are limited to “hundreds” of deaths and injured (Azerbaijan Supreme Court 2005).

5 According to the only source (in Russian) citing women’s involvement in direct combat, around 100 female soldiers fought in the conflict (Oganian and Mkhitarian 2001, p. 246). It is unclear whether the figure includes Armenian-Karabakhi women only or also ethnic Azerbaijani. Differently from male deaths, killed or injured women in this conflict have been described as “invisible victims” (Twum et al. 2019).

6 92% of all sampled women were ethnic Azerbaijani. 94% of women retained in the analytical sample (see below for details) were ethic Azerbaijani and 99% spoke Azerbaijani as native language (Table B1).

7 Specifically: “Is [NAME] an internally displaced person?” and “Is [NAME] a refugee?”.

8 Evidently, this strategy captures only any underreporting of women living with their origin, conflict-affected families in 2006, not those living outside (e.g., with partners). While I cannot rule this out, evidence suggests that even when IDP women marry non-IDP men, they rarely forgo their status because of its associated social protection benefits, and allegedly as a preventative measure against divorce (ICG, 2005).

9 It was not possible to obtain a list of district names matching numerical codes due to confidentiality.

10 Among observable characteristics in the analytic sample, the only significant differences were in urban/rural residence and household wealth, with non-migrant conflict-affected women being more likely to reside in rural areas and poorer. This is expected given that IDPs/refugees clustered in urban areas and often relied on dedicated state financial support.

11 Additional supporting information on the empirical strategy and assumption checks are in Appendix B (Tables B1–B3). Before survival models, I performed linear probability models including an interaction between *War-cohort* and each conflict indicator on a set of dependent variables indicating the probability of marriage by each age from 15-19 on both the full sample and on samples of those at risk of each outcome by 1996. Results (not presented for space reasons) show no significant differences, except at age 19, for conflict-affected women born 1974-1984, although the relationship is negative at most cut-offs.

12 Findings were robust to other cohort groupings, e.g., single-, two- and four-years. Statistically significant negative associations effectively characterised conflict-affected women born in 1975, 1978-1980 and 1982.

13 When I further disaggregated the variable to differentiate between refugees and IDPs, results did not change substantively. Refugees from Armenia born in 1978–1980 appeared to be the most sensitive to declining risk of teen union, but caution against interpreting the estimates is warranted given small sample.

14 Due to high politicisation of the conflict, data are insufficient to test whether a conflict-caused decline in sex-ratio in districts of Upper-Karabakh was related to the lower marriage probability of these, and other conflict-affected women. Pre-conflict (USSR) district-level census data are not available disaggregated by age, sex (and ethnicity) nor are post-independence population data. Even if post-conflict measures were possible to construct with the AZ-DHS or 1995 Living Standard Survey, these would be hardly useful in the absence of pre-conflict information on district-level age and sex structure.

15 I examined male marriage outcomes using a subsample of respondents selected for the AZ-DHS men’s questionnaire (N = 1,941). I observed neither differences in marital timing between men born in the *Soviet* and *War*-cohorts (even when interacted with region indicators to examine differences across areas with larger influx of IDPs/refugees) nor between those affected and not-affected by the conflict. This observation perhaps suggests marriage-market preference for non-affected women.

## Code Availability

All codes for data cleaning and analysis associated with the current submission are available from the author in STATA format upon request.
