# Supplementary material for: Wedding Amidst War? Armed Conflict and Female Teen Marriage in Azerbaijan
Source: Eur J Popul. 2022 Oct 31;38(5):1243–75. doi: 10.1007/s10680-022-09645-0 (PMC9727014; doi:10.1007/s10680-022-09645-0)
Supplement: Supplementary file 1 — Supplementary file1 (DOCX 316 KB) [file 10680_2022_9645_MOESM1_ESM.docx]

# APPENDIX A (ELECTRONIC SUPPLEMENTARY MATERIAL)

Appendix A contains supplementary material detailing (a) definitions of conflict measures used in the text, (Table A1); (b) identification of the conflict and non-conflict (cohorts (Table A2), (c) descriptive statistics of the analytical sample (Table A3), models by conflict-migration status (Table A4).

| **Group** | ***N*/%**  **(weighted)** | **Binary indicator** | **Continuous frequency indicator** | **Continuous intensity indicator** |
| --- | --- | --- | --- | --- |
| **Panel A: Cohorts** |  |  |  |  |
| Born 1974-1984  (aged 12-19 between 1992-1996) | 2,542 42.28% | 1 “War-cohort” |  |  |
|  |  |  |  |  |
| Born 1957-1971  (aged 21+ in 1992) | 3,469 57.72% | 0 “Soviet cohort” |  |  |
|  |  |  |  |  |
| **Panel B: Conflict-affectedness** |  |  |  |  |
| IDPs from Nagorno-Karabakh;  Non-refugees/IDPs with one displaced male member (or the mother, if household head) in origin family | 301  5.00% | 1 “Conflict-affected ” | Number of conflict events in 1992-1996 in district of origin | Number of conflict fatalities in 1992-1996 in  district of origin per 1,000 population (as of the 1989 USSR Population Census figures, *omitted thereafter*) |
|  |  |  |  |  |
| Refugees from Armenia | 168  2.79% | 1 “Conflict-affected ” | Mean number of conflict events in 1992-1996 in all Nagorno-Karabakh districts | Mean number of conflict fatalities in 1992-1996  in all Nagorno-Karabakh districts per 1,000 population |
|  |  |  |  |  |
| Permanent residents in contested districts (including non IDP/refugees migrating there during conflict) | 175  2.91% | 1 “Conflict-affected ” | Mean number of conflict events in  1992 (or year of arrival for migrants)-1996 in contested districts (Agdam, Terter and Fizuli) | Mean number of conflict fatalities in  1992 (or year of arrival for migrants)-1996 in contested districts (Agdam, Terter and Fizuli) per 1,000 population |
|  |  |  |  |  |
| All other women | 5,367 89.30% | 0 “Not-affected” | None | None |

#### Table A1 Definitions of conflict measures

Notes: In Panel A, the table shows cohort grouping for women who attained their teen ages between 1992-1996. In Panel B, it first shows the different groups making up the binary “overall conflict-affectedness” indicator, their counts and relative percentages in the analytic sample. It then shows how the continuous indicators were calculated for each group, in the absence of specific information on the district of residence for some groups (e.g., for refugees from Armenia) and considering their different locations during the conflict.

#### Table A2 Table of women’s attained ages during conflict by birth cohort

|  | **Age in 1992** | **Age in 1996** | **Age in 2006** | **Year woman attains age 12** | **Year woman attains age 19** | **Weighted** |
| --- | --- | --- | --- | --- | --- | --- |
|  |  |  |  |  |  | ***N*** |
| 1957 | 35 | 39 | 49 | 1969 | 1976 | 131 |
| 1958 | 34 | 38 | 48 | 1970 | 1977 | 195 |
| 1959 | 33 | 37 | 47 | 1971 | 1978 | 199 |
| 1960 | 32 | 36 | 46 | 1972 | 1979 | 236 |
| 1961 | 31 | 35 | 45 | 1973 | 1980 | 274 |
| 1962 | 30 | 34 | 44 | 1974 | 1981 | 257 |
| 1963 | 29 | 33 | 43 | 1975 | 1982 | 254 |
| 1964 | 28 | 32 | 42 | 1976 | 1983 | 265 |
| 1965 | 27 | 31 | 41 | 1977 | 1984 | 260 |
| 1966 | 26 | 30 | 40 | 1978 | 1985 | 250 |
| 1967 | 25 | 29 | 39 | 1979 | 1986 | 220 |
| 1968 | 24 | 28 | 38 | 1980 | 1987 | 233 |
| 1969 | 23 | 27 | 37 | 1981 | 1988 | 211 |
| 1970 | 22 | 26 | 36 | 1982 | 1989 | 250 |
| 1971 | 21 | 25 | 35 | 1983 | 1990 | 235 |
| 1972 | 20 | 24 | 34 | 1984 | 1991 | 197 |
| 1973 | 19 | 23 | 33 | 1985 | 1992 | 206 |
| **1974** | **18** | 22 | 32 | **1986** | **1993** | **203** |
| **1975** | **17** | 21 | 31 | **1987** | **1994** | **185** |
| **1976** | **16** | 20 | 30 | **1988** | **1995** | **213** |
| **1977** | **15** | **19** | 29 | **1989** | **1996** | **203** |
| **1978** | **14** | **18** | 28 | **1990** | **1997** | **205** |
| **1979** | **13** | **17** | 27 | **1991** | **1998** | **196** |
| **1980** | **12** | **16** | 26 | **1992** | **1999** | **264** |
| **1981** | 11 | **15** | 25 | **1993** | **2000** | **220** |
| **1982** | 10 | **14** | 24 | **1994** | **2001** | **291** |
| **1983** | 9 | **13** | 23 | **1995** | **2002** | **305** |
| **1984** | 8 | **12** | 22 | **1996** | **2003** | **256** |
| Overall Total | 8-35 | 12-39 | 22-49 |  |  | 6,414 |

Source: 2006 AZ-DHS.

Notes: *N* indicates the total number of women in the sample (including women born 1972-1973) weighted using provided sample weights. In bold are birth cohorts of women who reached teen ages (12-19) between 1992-1996 and hence were ‘at risk’ of teen union during the peak years of the Nagorno-Karabakh conflict.

#### Table A3 Descriptive statistics of the weighted sample used in the analyses

|  | ***N*** | **Mean or %** | ***s.d.*** | **Min** | **Max** | **Person-years** |
| --- | --- | --- | --- | --- | --- | --- |
|  |  |  |  |  |  |  |
| **Conflict frequency (events)** | 6,011 | 1.97 | 6.69 | 0 | 73 | 44,885 |
| **Conflict intensity (fatalities per 1,000)** | 6,011 | 0.56 | 2.48 | 0 | 66.38 | 44,885 |
| **Age** | 6,011 | 35.08 | 8.36 | 21 | 49 | 44,885 |
|  |  |  |  |  |  |  |
| **Overall conflict affectedness** | |  |  |  |  |  |
| Non-affected | 5,367 | 89.30% |  |  |  | 40,062 |
| Conflict-affected | 644 | 10.70% |  |  |  | 4,823 |
|  |  |  |  |  |  |  |
| **Cohort** |  |  |  |  |  |  |
| 1957-1959 | 525 | 8.73% |  |  |  | 3,921 |
| 1960-1962 | 767 | 12.76% |  |  |  | 5,857 |
| 1963-1965 | 779 | 12.95% |  |  |  | 5,901 |
| 1966-1968 | 702 | 11.69% |  |  |  | 5,296 |
| 1969-1971 | 696 | 11.59% |  |  |  | 5,309 |
| 1974-1977 | 804 | 13.37% |  |  |  | 5,787 |
| 1978-1980 | 665 | 11.06% |  |  |  | 4,827 |
| 1981-1984 | 1,073 | 17.85% |  |  |  | 7,987 |
|  |  |  |  |  |  |  |
| **Conflict cohort** |  |  |  |  |  |  |
| 1957-1971 | 3,469 | 57.72% |  |  |  | 26,280 |
| 1974-1984 | 2,542 | 42.28% |  |  |  | 18,605 |
|  |  |  |  |  |  |  |
| **Residence type** |  |  |  |  |  |  |
| Rural | 2,599 | 43.75% |  |  |  | 19,205 |
| Urban | 3,412 | 56.25% |  |  |  | 25,680 |
|  |  |  |  |  |  |  |
|  |  |  |  |  |  |  |
| **Married in teen ages** |  |  |  |  |  |  |
| Yes | 1,846 | 30.71% |  |  |  | 11,572 |
| No | 4,165 | 69.29% |  |  |  | 33,313 |
|  |  |  |  |  |  |  |
| **Married by 15** |  |  |  |  |  |  |
| Yes | 104 | 1.69% |  |  |  | - |
| No | 5,907 | 98.31% |  |  |  | - |
|  |  |  |  |  |  |  |
| **Married by 16** |  |  |  |  |  |  |
| Yes | 280 | 4.64% |  |  |  | - |
| No | 5,731 | 95.36% |  |  |  | - |
|  |  |  |  |  |  |  |
| **Married by 17** |  |  |  |  |  |  |
| Yes | 675 | 11.19% |  |  |  | - |
| No | 5,336 | 88.81% |  |  |  | - |
|  |  |  |  |  |  |  |
| **Married by 18** |  |  |  |  |  |  |
| Yes | 1,229 | 20.32% |  |  |  | - |
| No | 4,782 | 79.68% |  |  |  | - |
|  |  |  |  |  |  |  |
| Source: 2006 AZ-DHS.  Notes: All indicators are presented using provided sample weights. | | | | | | |

#### Table A4 Results of discrete-time clog-log models of the transition to teen marriage with granular cohorts and migration status

|  | **HR of teen union** |
| --- | --- |
|  | (1) |
|  | |
| **Conflict migration status * Born in** | |
| IDP/refugee * |  |
| 1974-1977 | 0.810 |
|  | [0.44,1.49] |
| 1978-1980 | 0.331** |
|  | [0.15,0.73] |
| 1981-1984 | 0.693 |
|  | [0.37,1.28] |
| Non-migrant in Upper-Karabakh * |  |
| 1974-1977 | 1.362 |
|  | [0.55,3.37] |
| 1978-1980 | 0.256* |
|  | [0.25,0.98] |
| 1981-1984 | 0.182* |
|  | [0.04,0.87] |
|  |  |
| **Overall conflict-affectedness (ref: Not-affected)** |  |
| IDP/refugee | 1.323 |
|  | [0.693,2.52] |
| Non-migrant in Upper-Karabakh | 1.163 |
|  | [0.72,1.88] |
|  |  |
| **District dummies** | Yes |
| **Year of birth dummies** | Yes |
| **Controls** | Yes |
| **σ_u_^2^** | 1.169 |
| ***N person-years*** | 44,885 |
| Source: 2006 AZ-DHS.  Notes: Sample consists of women born during 1957–1984 (ages 22–49 in 2006), excluding women born 1972-1973. Subjects enter analysis at age 12. Columns represent hazard ratios. 95% confidence intervals are in parentheses. Robust standard errors clustered at the PSU level. The “War-cohort” includes women born 1974-1984. Non-migrants in Upper-Karabakh include women who always resided (or migrated pre-conflict or during conflict) in the Azerbaijani controlled parts of the contested districts of Agdam, Fizuli and Terter. All regressions control for duration since start of exposure to the risk of teen marriage (<5 years, 5-6 years and >6 years) and rural/urban residence, and include a constant not shown. Models are specified with individual-level frailty terms (σ_u_^2^) and are weighted using provided sampling weights. * p<0.05, ** p<0.01, *** p<0.001. | |

#### Fig A1 Yearly conflict events and fatalities, Azerbaijan 1992-2006

Source*:* UCDP-GED (2021).

Notes: Darker bars and thicker dashed line highlight events and fatalities in peak conflict years. The y-axis on left-hand side refers to conflict events, the y-axis on the right-hand side to the number of reported fatalities.

####
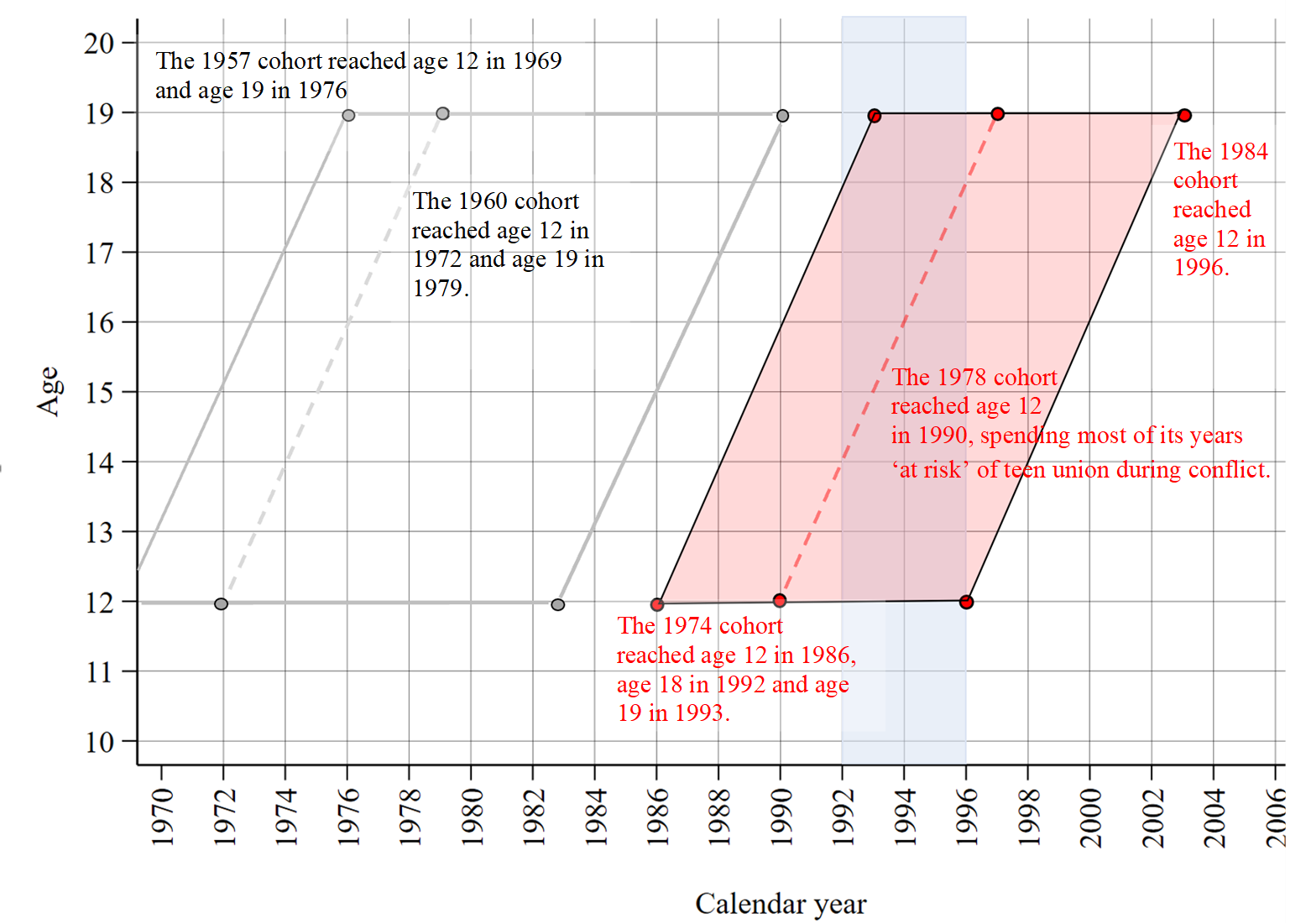
Fig A2 Lexis visualisation of women’s attained ages during conflict years

Notes: The blue rectangle shows the core conflict years (1992-1996). The grey parallelogram indicates the ‘Soviet cohort’ (born 1957-1971), i.e., women who were 21+ before conflict onset. These women were too old to have their teen marriage outcomes affected by the conflict. The red parallelogram highlights attained ages in each calendar period for the ‘War-cohorts’ (1974-1984), i.e., for women who turned 12-19 during the core conflict years. As explained in the text, the cohorts aged 19-20 in 1992 (born 1973-1972) are excluded in the main analyses as their “conflict-affectedness” status is less clear-cut.

# APPENDIX B (ELECTRONIC SUPPLEMENTARY MATERIAL)

Appendix B contains supplementary material detailing (a) assumption checks in support to the difference-in-difference *logic* applied in the analyses, including balance of covariates and placebo test (Tables B1-B3); (b) results from alternative model specifications, including alternative cohort grouping (Tables B4-B6) and conflict measures (Table B7).

## Preliminary checks

### Balance in War- and Soviet-cohort characteristics

A preliminary concern is the possibility that older and younger cohorts differ systematically and in relation to the conflict (selection into treatment). One way of testing this aspect would be analysing whether the origin households of slightly younger and slightly older women were similar in terms of various characteristics, including conflict-affectedness. Unfortunately, the AZ-DHS offers limited information on women’s natal household and does so only for those – whether married or not – who were still living with their origin families at survey time. These are predominantly the youngest respondents. Alternatively, one can look at whether all younger and older women are similar at least in terms of observable characteristics. I follow both approaches for completeness. Table B1 shows the distribution or means of several observable characteristics for women in the *Soviet* vs. *War-cohorts*, while Table B2 for women still living with their origin household (*N*=1,274).

There is substantial balance across covariates between the *Soviet cohort* and the *War-cohort*, regardless of whether they still live with their origin family. In Table B1, the only significant difference is observed in the age variable which is expected by default. This finding strengthens the interpretability of results. In Table B2 (women still residing with their natal household in 2006), the only other notable difference is in household wealth, which favours the *War-cohort*. This observation might suggest that girls in the *War-cohort* were somehow wealthier than older cohorts and thus, if affected by the conflict, their families could afford keeping them within the household rather than “cashing them in” to prospective husbands. However, these covariates were all measured in 2006 and there is no way to know whether responses for characteristics like wealth changed over time and compared to the conflict years. Furthermore, women still residing with their origin family at survey time, especially older ones, are likely to be a selected group and hence not necessarily displaying an accurate pattern. For these reasons, I do not control for household wealth in the main models. Even when included, results do not change substantively in all model specifications.

#### Table B1 Descriptive statistics of Soviet and War-cohorts

|  | **Cohort** | | | | |
| --- | --- | --- | --- | --- | --- |
|  | **1957-1971 (“Soviet”)** | | **1974-1984 (“War”)** | |  |
|  | *N* | Mean or % | *N* | Mean or % | *p-value* |
|  |  |  |  |  |  |
| **Conflict frequency (events)** | 3,469 | 1.96 | 2,542 | 1.99 |  |
| **Conflict intensity (fatalities per 1,000)** | 3,469 | 0.54 | 2,542 | 0.59 |  |
| **Overall conflict-affectedness** |  |  |  |  |  |
| Non-affected | 3,107 | 88.56% | 2,260 | 88.94% |  |
| Conflict-affected | 362 | 10.44% | 282 | 11.06% |  |
|  |  |  |  |  |  |
| **Age** | 3,469 | 41.48 | 2,542 | 26.35 | *** |
| **Years of education** | 3,469 | 10.88 | 2,542 | 11.00 |  |
|  |  |  |  |  |  |
| **Household wealth** |  |  |  |  |  |
| Poor | 1,336 | 38.50% | 947 | 37.26% |  |
| Middle | 674 | 19.44% | 534 | 21.00% |  |
| Rich | 1,459 | 42.06% | 1,061 | 41.74% |  |
|  |  |  |  |  |  |
| **Residence type** |  |  |  |  |  |
| Rural | 1,497 | 43.17% | 1,103 | 43.36% |  |
| Urban | 1,972 | 56.83% | 1,439 | 56.64% |  |
|  |  |  |  |  |  |
| **Ethnicity** |  |  |  |  |  |
| Azerbaijani | 3,256 | 93.85% | 2,397 | 94.28% |  |
| Talish | 67 | 1.92% | 40 | 1.59% |  |
| Lesgin | 48 | 1.40% | 38 | 1.48% |  |
| Russian | 22 | 0.63% | 5 | 0.21% |  |
| Other | 76 | 2.20% | 62 | 2.44% |  |
|  |  |  |  |  |  |
| **Religion** |  |  |  |  |  |
| Muslim | 3,435 | 99.01% | 2,531 | 99.24% |  |
| Other | 34 | 0.99% | 11 | 0.76% |  |
|  |  |  |  |  |  |
| **Married in teen ages** |  |  |  |  |  |
| Yes | 2,478 | 14.33% | 855 | 24.38% | *** |
| No | 991 | 85.67% | 1,687 | 75.62% |  |
|  |  |  |  |  |  |
| **Married by 15** |  |  |  |  |  |
| Yes | 32 | 0.90% | 73 | 2.85% | *** |
| No | 3,437 | 99.10% | 2,469 | 97.15% |  |
|  |  |  |  |  |  |
| **Married by 16** |  |  |  |  |  |
| Yes | 76 | 2.16% | 206 | 8.05% | *** |
| No | 3,395 | 97.84% | 2,336 | 91.95% |  |
|  |  |  |  |  |  |
| **Married by 17** |  |  |  |  |  |
| Yes | 274 | 7.90% | 401 | 15.76% | *** |
| No | 3,195 | 92.10% | 2,141 | 84.24% |  |
|  |  |  |  |  |  |
| **Married by 18** |  |  |  |  |  |
| Yes | 618 | 17.82% | 611 | 24.00% | *** |
| No | 2,851 | 82.18% | 1,931 | 76.00% |  |
|  |  |  |  |  |  |

Source: 2006 AZ-DHS.

Notes: All indicators are presented using provided sample weights. * p<0.05, ** p<0.01, *** p<0.001.

#### Table B2 Descriptive characteristics of women of Soviet and War-cohorts living with their origin households in 2006

| **Cohort** | | | | | |
| --- | --- | --- | --- | --- | --- |
|  | **1957-1971 (“Soviet”)** | | **1974-1984 (“War”)** | |  |
|  | *N* | Mean or % | *N* | Mean or % | *p-value* |
|  |  |  |  |  |  |
| **Conflict frequency (events)** | 424 | 2.09 | 850 | 1.74 |  |
| **Conflict intensity (fatalities per 1,000)** | 424 | 0.57 | 850 | 0.53 |  |
| **Overall conflict-affectedness** |  |  |  |  |  |
| Non-affected | 377 | 88.82% | 761 | 89.53% |  |
| Conflict-affected | 47 | 11.18% | 89 | 10.47% |  |
|  |  |  |  |  |  |
| **Age** | 424 | 39.78 | 850 | 25.31 | *** |
| **Years of education** | 424 | 10.71 | 850 | 11.51 |  |
| **Number of household members** | 424 | 5.52 | 850 | 5.37 |  |
|  |  |  |  |  |  |
| **Household wealth** |  |  |  |  | ** |
| Poor | 204 | 48.06% | 284 | 33.43% |  |
| Middle | 73 | 17.23% | 173 | 20.38% |  |
| Rich | 147 | 34.71% | 393 | 46.18% |  |
|  |  |  |  |  |  |
| **Residence type** |  |  |  |  |  |
| Rural | 219 | 48.44% | 499 | 41.20% |  |
| Urban | 205 | 51.56% | 351 | 58.80% |  |
|  |  |  |  |  |  |
| **Ethnicity** |  |  |  |  |  |
| Azerbaijani | 395 | 92.96% | 802 | 94.32% |  |
| Talish | 7 | 1.70% | 12 | 1.43% |  |
| Russian | 8 | 1.90% | 2 | 0.20% |  |
| Other | 14 | 3.44% | 34 | 4.05% |  |
|  |  |  |  |  |  |
| **Religion** |  |  |  |  |  |
| Muslim | 415 | 97.86% | 845 | 99.38% |  |
| Other | 9 | 2.14% | 5 | 0.62% |  |
|  |  |  |  |  |  |
| **Married in teen ages** |  |  |  |  |  |
| Yes | 39 | 9.14% | 74 | 8.76% |  |
| No | 385 | 90.86% | 776 | 91.24% |  |
|  |  |  |  |  |  |
| **Married by 15** |  |  |  |  |  |
| Yes | 3 | 0.71% | 10 | 1.13% |  |
| No | 421 | 99.29% | 840 | 98.87% |  |
|  |  |  |  |  |  |
| **Married by 16** |  |  |  |  |  |
| Yes | 6 | 1.48% | 30 | 3.58% |  |
| No | 418 | 98.52% | 820 | 96.42% |  |
|  |  |  |  |  |  |
| **Married by 17** |  |  |  |  |  |
| Yes | 9 | 2.10% | 45 | 5.23% | * |
| No | 415 | 97.90% | 805 | 94.77% |  |
|  |  |  |  |  |  |
| **Married by 18** |  |  |  |  |  |
| Yes | 19 | 4.44% | 57 | 6.66% |  |
| No | 405 | 95.56% | 793 | 93.34% |  |
|  |  |  |  |  |  |

Source: 2006 AZ-DHS.

Notes: All indicators are presented using provided sample weights. * p<0.05, ** p<0.01, *** p<0.001

### Placebo test

Figure 3 in the main text provides suggestive evidence that trends in teen marriage would have been the same for affected and non-affected in the absence of conflict. As an additional test, I follow Valente (2011) and estimate Equation (1) replacing the *War-Cohort*_ik_ indicator in the interaction term with a set of dummies for the five youngest cohorts who, due to their year of birth, could not have had their teen marriage patterns affected by conflict (1967≤ *k*≤1971). These models are then estimated on the sample of the oldest ten *Soviet cohorts* i.e., women born between 1961-1971.^^[[1]](#footnote-1)^^ The interaction terms between each of the five youngest cohorts and each conflict indicators are not jointly nor individually significant, suggesting no systematic differences in pre-conflict early marriage trends between groups with *future* different exposure to the conflict. Additionally, these results help dismissing the possibility that the main findings are driven by events occurred before conflict onset. Table B3 reports the full results of this “placebo” experiment.

| **Sample: Born 1961-1971** |  | | |
| --- | --- | --- | --- |
|  | **Overall conflict-affectedness** | **Conflict frequency (events)** | **Conflict intensity (fatalities per 1,000)** |
| **Age in 1992 * Conflict measure** | |  |  |
| 25 (born 1967) * Conflict measure | 0.97 [0.41,2.30] | 1.01 [0.97,1.05] | 1.01 [0.87,1.18] |
| 24 (born 1968) * Conflict measure | 0.85 [0.29,2.47] | 0.99 [0.94,1.04] | 0.96 [0.79,1.16] |
| 23 (born 1969) * Conflict measure | 0.84 [0.29,2.43] | 0.99 [0.95,1.05] | 0.98 [0.82,1.17] |
| 22 (born 1970) * Conflict measure | 1.68 [0.72,3.93] | 1.02 [0.98,1.06] | 1.08 [0.94,1.24] |
| 21 (born 1971) * Conflict measure | 0.58 [0.22,1.56] | 0.97 [0.94,1.04] | 0.95 [0.80,1.14] |
| **District dummies**^[[2]](#footnote-2)^ | Yes | Yes | Yes |
| **Year of birth dummies** | Yes | Yes | Yes |
| ***N*** **Person-years** | 20,597 | 20,597 | 20,597 |
| **F-test pre-conflict trend difference** | 0.658 | 0.720 | 0.836 |
| Source: 2006 AZ-DHS.  Notes: Sample consists of women born 1961–1971 (ages 35–45 in 2006 and 21–31 at the start of the conflict in 1992). Columns represent hazard ratios. 95% confidence intervals are in parentheses. Robust standard errors clustered at PSU level are in parentheses. Reference category for the cohort measure is “Born in 1961-1966”. The binary indicator “overall conflict-affectedness” is equal to “1” for IDP/refugee women, non-migrant women residing in Upper-Karabakh and non-displaced women with at least one member of their family of origin who identified as IDP/refugee, and “0” otherwise. All regressions are specified with frailty terms (σ_u_^2^) at the individual level. Models control for duration since start of exposure to the risk of teen marriage (<5 years, 5-6 years and >6 years) and rural/urban residence, and include a constant not shown. Subjects enter analysis at age 12. * p<0.05, ** p<0.01, *** p<0.001. | | | |

#### Table B3 Test for pre-conflict difference in early marriage

#### Table B4 Results of discrete-time clog-log models of the transition to teen marriage using alternative (restricted) control group

|  | **HR of teen union** | | |
| --- | --- | --- | --- |
|  | (1) | (2) | (3) |
|  |  |  |  |
| **War-cohort (1974-1984) * Conflict measure** | 0.565* | 0.979* | 0.931* |
|  | [0.37,0.87] | [0.96,0.99] | [0.87,0.99] |
| **Overall conflict-affectedness (ref: Not-affected)** |  |  |  |
| Conflict-affected | 1.465 |  |  |
|  | [0.81,2.63] |  |  |
| **Conflict frequency (events)** |  | 1.037* |  |
|  |  | [1.01.1.07] |  |
| **Conflict intensity (fatalities per 1,000)** |  |  | 1.091 |
|  |  |  | [0.94,1.27] |
|  |  |  |  |
| **District dummies** | Yes | Yes | Yes |
| **Year of birth dummies** | Yes | Yes | Yes |
| **Controls** | Yes | Yes | Yes |
| **σ_u_^2^** | 1.100 | 1.104 | 0.740 |
| ***N person-years*** | 38,934 | 38,934 | 38,934 |
| Source: 2006 AZ-DHS.  Notes: Sample consists of women born 1961–1984 (ages 22–45 in 2006), excluding women born 1972-1973. Subjects enter analysis at age 12. Columns represent hazard ratios. 95% confidence intervals are in parentheses. Robust standard errors clustered at the PSU level. The “War-cohort” includes women born 1974-1984. The binary indicator “overall conflict-affectedness” is equal to “1” for IDP/refugee women, non-migrant women residing in Upper-Karabakh and non-displaced women with at least one member of their family of origin who identified as IDP/refugee, and “0” otherwise. All regressions control for duration since start of exposure to the risk of teen marriage (<5 years, 5-6 years and >6 years) and rural/urban residence, and include a constant not shown. Models are specified with individual-level frailty terms (σ_u_^2^) and are weighted using provided sampling weights. * p<0.05, ** p<0.01, *** p<0.001. | | | |

#### Table B5 Results of discrete-time clog-log models of the transition to teen marriage including 1972-1973 cohorts (both coded as ‘Soviet cohorts’)

|  | **HR of teen union** | | |
| --- | --- | --- | --- |
|  | (1) | (2) | (3) |
|  |  |  |  |
| **War-cohort (1974-1984) * Conflict measure** | 0.560** | 0.979** | 0.928* |
|  | [0.55,0.81] | [0.96,0.98] | [0.87,0.99] |
| **Overall conflict-affectedness (ref: Not-affected)** |  |  |  |
| Conflict-affected | 1.333 |  |  |
|  | [0.81,2.20] |  |  |
| **Conflict frequency (events)** |  | 1.037* |  |
|  |  | [1.01,1.07] |  |
| **Conflict intensity (fatalities per 1,000)** |  |  | 1.104 |
|  |  |  | [0.98,1.24] |
|  |  |  |  |
| **District dummies** | Yes | Yes | Yes |
| **Year of birth dummies** | Yes | Yes | Yes |
| **Controls** | Yes | Yes | Yes |
| **σ_u_^2^** | 0.796 | 1.125 | 0.668 |
| ***N person-years*** | 47,960 | 47,960 | 47,960 |
| Source: 2006 AZ-DHS.  Notes: Sample consists of women born 1957–1984 (ages 22–49 in 2006), including women born 1972-1973. Subjects enter analysis at age 12. Columns represent hazard ratios. 95% confidence intervals are in parentheses. Robust standard errors clustered at the PSU level. The “War-cohort” includes women born 1974-1984. The binary indicator “overall conflict-affectedness” is equal to “1” for IDP/refugee women, non-migrant women residing in Upper-Karabakh and non-displaced women with at least one member of their family of origin who identified as IDP/refugee, and “0” otherwise. All regressions control for duration since start of exposure to the risk of teen marriage (<5 years, 5-6 years and >6 years) and rural/urban residence, and include a constant not shown. Models are specified with individual-level frailty terms (σ_u_^2^) and are weighted using provided sampling weights. * p<0.05, ** p<0.01, *** p<0.001. | | | |

#### Table B6 Results of discrete-time clog-log models of the transition to teen marriage including 1972-1973 cohorts (1973 coded as ‘War-cohort’)

|  | **HR of teen union** | | |
| --- | --- | --- | --- |
|  | (1) | (2) | (3) |
|  |  |  |  |
| **War-cohort (1973-1984) * Conflict measure** | 0.690* | 0.985* | 0.948* |
|  | [0.47,0.98] | [0.97,0.99] | [0.89,0.99] |
| **Overall conflict-affectedness (ref: Not-affected)** |  |  |  |
| Conflict-affected | 1.243 |  |  |
|  | [0.75,2.05] |  |  |
| **Conflict frequency (events)** |  | 1.035* |  |
|  |  | [1.01,1.07] |  |
| **Conflict intensity (fatalities per 1,000)** |  |  | 1.098 |
|  |  |  | [0.98,1.23] |
|  |  |  |  |
| **District dummies** | Yes | Yes | Yes |
| **Year of birth dummies** | Yes | Yes | Yes |
| **Controls** | Yes | Yes | Yes |
| **σ_u_^2^** | 1.127 | 1.144 | 1.093 |
| ***N person-years*** | 47,960 | 47,960 | 47,960 |
| Source: 2006 AZ-DHS.  Notes: Sample consists of women born during 1957–1984 (ages 22–49 in 2006), including women born 1972-1973. Subjects enter analysis at age 12. Columns represent hazard ratios. 95% confidence intervals are in parentheses. Robust standard errors clustered at the PSU level. The “War-cohort” includes women born 1974-1984. The binary indicator “overall conflict-affectedness” is equal to “1” for IDP/refugee women, non-migrant women residing in Upper-Karabakh and non-displaced women with at least one member of their family of origin who identified as IDP/refugee, and “0” otherwise. All regressions control for duration since start of exposure to the risk of teen marriage (<5 years, 5-6 years and >6 years) and rural/urban residence, and include a constant not shown. Models are specified with individual-level frailty terms (σ_u_^2^) and are weighted using provided sampling weights. * p<0.05, ** p<0.01, *** p<0.001. | | | |

#### Table B7 Results of discrete-time clog-log models of the transition to teen marriage using categorical conflict frequency and intensity indicators

|  | **HR of teen union** | |
| --- | --- | --- |
|  | (1) | (2) |
|  |  |  |
| **War-cohort (1974-1984) * Conflict frequency (events)** |  |  |
| Medium (<25) | 0.641* [0.41,0.98] |  |
| High (25+) | 0.619 [0.25,1.50] |  |
|  |  |  |
| **Number of conflict events** |  |  |
| Medium (<25) | 1.571 [0.74,3.34] |  |
| High (25+) | 1.006 [0.37,2.73] |  |
|  |  |  |
|  |  |  |
| **War-cohort (1974-1984) * Conflict frequency (fatalities per 1,000)** | |  |
| Medium (<6 per 1,000) |  | 0.544* [0.28,0.94] |
| High (6+ per 1,000) |  | 0.852* [0.49,0.97] |
|  |  |  |
| **Number of conflict fatalities** |  |  |
| Medium |  | 0.883 [0.53,1.92] |
| High |  | 1.836 [0.98,3.45] |
|  |  |  |
| **District dummies** | Yes | Yes |
| **Year of birth dummies** | Yes | Yes |
| **Controls** | Yes | Yes |
| **σ_u_^2^** | 1.346 | 1.237 |
| ***N person-years*** | 44,885 | 44,885 |
| Source: 2006 AZ-DHS.  Notes: Reference categories: “No conflict events”, “No conflict fatalities”. “Medium” (between 1 and the 95^th^ percentile) and “High” (above the 95^th^ percentile). Sample consists of women born during 1957–1984 (ages 22–49 in 2006), excluding women born 1972-1973. Subjects enter analysis at age 12. Columns represent hazard ratios. 95% confidence intervals are in parentheses. Robust standard errors clustered at the PSU level. Conflict-affectedness and War-cohort variables as per Data and measures section. All regressions control for dummies since start of exposure (<5 years, 5-6 years and >6 years) and rural/urban residence, and include a constant not shown. Models are specified with individual-level frailty terms (σu2) and are weighted using provided sampling weights. * p<0.05, ** p<0.01, *** p<0.001. | | |

1. Models run on the full counterfactual sample (born 1957-1971) yielded similar estimates. [↑](#footnote-ref-1)
2. Models estimated without district dummies yielded similar estimates (available upon request). [↑](#footnote-ref-2)
